# The Rice Malectin Regulates Plant Cell Death and Disease Resistance by Participating in Glycoprotein Quality Control

**DOI:** 10.3390/ijms23105819

**Published:** 2022-05-22

**Authors:** Huijing Feng, Tiancheng Qiu, Changfa Yin, Xiaosheng Zhao, Guangyuan Xu, Linlu Qi, Yan Zhang, Youliang Peng, Wensheng Zhao

**Affiliations:** 1 State Key Laboratory of Agrobiotechnology, Beijing 100193, China; 2 MOA Key Lab of Pest Monitoring and Green Management, College of Plant Protection, China Agricultural University, Beijing 100193, China; fenghj0425@163.com (H.F.); qtc303@163.com (T.Q.); yinchangfa@cau.edu.cn (C.Y.); zxs718@126.com (X.Z.); xuguangyuan@cau.edu.cn (G.X.); qilinlu1987@126.com (L.Q.); yzhang@cau.edu.cn (Y.Z.); pengyl@cau.edu.cn (Y.P.)

**Keywords:** cell death, disease resistance, ER quality control, *Oryza sativa*, malectin, ribophorin I, OsSERK1, OsSERK2

## Abstract

In animals, malectin is well known to play an essential role in endoplasmic reticulum quality control (ERQC) by interacting with ribophorin I, one unit of the oligosaccharyltransferase (OST) complex. However, the functions of malectin in plants remain largely unknown. Here, we demonstrate the rice *OsMLD1* is an ER- and Golgi-associated malectin protein and physically interacts with rice homolog of ribophorin I (OsRpn1), and its disruption leads to spontaneous lesion mimic lesions, enhanced disease resistance, and prolonged ER stress. In addition, there are many more *N*-glycosites and *N*-glycoproteins identified from the *mld1* mutant than wildtype. Furthermore, OsSERK1 and OsSERK2, which have more *N*-glycosites in *mld1*, were demonstrated to interact with OsMLD1. *OsMLD1* can suppress OsSERK1- or OsSERK2-induced cell death. Thus, *OsMLD1* may play a similar role to its mammalian homologs in glycoprotein quality control, thereby regulating cell death and immunity of rice, which uncovers the function of malectin in plants.

## 1. Introduction

Plant programmed cell death (PCD) is a conserved pathway that is involved in multiple biological processes such as defense, development, and the stress response. The invasion of biotrophic or hemibiotrophic pathogens in plants can elicit pathogen-triggered PCD (pPCD), triggering a hypersensitive response (HR) at the site of the attempted attack [1]. In order to protect themselves from biotic stress, plants employed HR as a defense mechanism that usually is accompanied by the generation of reactive oxygen species (ROS), upregulation of pathogenesis-related (PR) proteins, accumulation of callose, and thickening of cell walls at the infection sites [2].

Lesion mimic mutants (LMMs), also called spotted leaf (*spl*) mutants, are considered as suitable materials for elucidating the mechanisms underlying plant responses to pathogen attacks and environmental stresses. LMMs exhibit spontaneous cell death similar to that caused by HR in the absence of pathogen infection, abiotic stress, or mechanical damage. In many cases, LMMs exhibit significantly enhanced disease resistance. To date, dozens of genes have been characterized from rice LMMs. The proteins contributing to the characteristics of LMMs are very diverse, such as transcription factors [3], oxidoreductases, ubiquitinations [4], zinc finger proteins [5], membrane-associated proteins [6], clathrin-associated adaptor proteins [7], ion channel proteins [8], nucleotide-binding site–leucine-rich repeat (NLR) proteins [9], mRNA splicing factors [10], porphyrin [11], protein kinases [12], UDP-N-acetylglucosamine pyrophosphorylase [13], AAA-type ATPase [14], eEF1A-like protein [15], eukaryotic release factor 1 protein [16], receptor-like kinases [17,18], CUE domain protein [19], ATP-citrate lyase [20], hydroperoxide lyase [21], and glycine-rich domain protein [22]. Such functional diverse proteins suggest complicated molecular mechanisms regulating defense responses in plants.

The endoplasmic reticulum (ER) is an organelle that plays a critical role in protein synthesis, folding, modification, and secretion. To maintain protein homeostasis inside the ER, plant cells are equipped with ER quality control (ERQC) to cope with increased amounts of unfolded or misfolded proteins. When organisms are subjected to biotic or abiotic stress, the levels of misfolded proteins may overwhelm the capacity of the ERQC system, resulting in ER stress [23,24,25]. To avoid such serious consequences, cells activate several processes within the unfolded protein response (UPR) to restore protein homeostasis within the ER, either through the enhancement of protein folding and degradation competence or by alleviating the demands for such reactions [25]. The UPR is, therefore, supposed to ensure cell survival. However, under severe or prolonged ER stress, an apoptosis-like programed cell death (ER-CD) is activated in mammalian cells to eliminate damaged cells from stressed organisms [26,27]. In plants, induction of UPR with tunicamycin treatment is followed by cell death [28,29,30]. Such an ER-induced PCD also occurred in plants attacked by pathogens, and this ER-induced PCD can be deployed by some microbes to establish colonization or infection [31,32,33].

In plants, there are at least two UPR pathways. One is mediated by IRE1–bZIP60 and the other is mediated by bZIP28 [34]. Previous studies showed that adapting ER folding capacity and UPR regulation play important roles in plant immunity. For example, the *Arabidopsis* pattern-recognition receptor EFR requires the ERQC complex SDF2–ERdj3B–BiP for proper processing [35,36]. Meanwhile, functions of a number of membrane-localized immune receptors depend on ERQC, including rice PRR XA21 [37,38], an induced receptor kinase (IRK) of tobacco [39], and glycosylated Cf proteins of tomato [40]. In addition, the IRE1–bZIP60 branch is crucial for inducing systemic acquired resistance (SAR) against bacterial pathogens and abiotic stress tolerance [41]. A membrane-associated transcription factor NAC089 has been reported to control ER stress-induced programmed cell death in plants. *NAC089* is upregulated by ER stress, which is directly controlled by bZIP28 and bZIP60 [42].

The majority of ER-targeted proteins are *N*-glycosylated [43]. *N*-Glycosylation plays versatile roles during protein folding. *N*-Glycosylation refers to the cotranslational and post-translational covalent attachment of an oligosaccharide (Glc3–Man9–GlcNAc2 in eukaryotes) in the N–X–S/T context (where X is not Pro) [44]. After attachment of the oligosaccharide (glycan) to a protein in the ER, it can be further modified in the secretory pathway. In animals, the glycoprotein can interact with the lectin malectin, a membrane-associated ER-resident protein that specifically recognizes *di*-glycosylated glycans [45]. This protein plays a role in the quality control of glycoproteins by forming a complex with ribophorin I for enhanced association with misfolded glycoproteins [46,47]. Ribophorin I is an ER-resident transmembrane protein that serves as a subunit of the mammalian OST complex and acts as a substrate-specific facilitator of *N*-glycosylation [48]. However, if and how the malectin participates in *N*-glycoprotein equality control in plants is still unknown. CrRLK1L members, known as malectin receptor-like kinases (MRLKs), were first discovered in *Catharanthus roseus* [49]. In *Arabidopsis*, members of the CrRLK1L subfamily each contain an extracellular malectin/malectin-like domain (MLD), a transmembrane domain, and a cytoplasmic kinase domain. The malectin domain sequences identified family members in the *A. thaliana* proteome have a single malectin domain or tandem malectin domains in the ECDs of protein kinases [50]. Studies have demonstrated that M/MLD-RLKs are crucial for plant growth and survival and regulate diverse processes, including pollen tube reception and tip growth, cell-wall integrity sensing, and hormonal responses. More importantly, mounting evidence has indicated a vital role for MLRs in plant immunity recently [51,52]. However, they are not thought to be involved in *N*-glycosylation owing to their plasma membrane localization and the weak conservation of residues mediating the interaction with glucose residues in the original malectin [53]. More recently, a tomato malectin (referred to as malectin-like domain protein, SlRLK-like, in the original publication), was shown to affect the localization and abundance of the LeEIX2 receptor, resulting in the suppression of EIX-induced immune responses in tomato [54]. Moreover, recent research has revealed that the MLD of the MLD-containing receptor IOS1 retains IOS1 in the ER of plant cells and attenuates the infection-induced UPR in *Arabidopsis thaliana* [55].

Here, we uncover the function of the malectin protein OsMLD1, which is associated with the ER and Golgi. Disruption of *OsMLD1* resulted in prolonged ER stress, activation of UPR, and enhanced disease resistance. *OsMLD1* physically interacts with the rice homolog of ribophorin I, OsRpn1, indicating that *OsMLD1* functions as a unit of the OST complex. *N*-Glycoproteome analysis showed there are many more *N*-glycosites and *N*-glycoproteins in the *mld1* mutant than in wildtype, suggesting that *OsMLD1* plays a role in protein glycosylation modification. Furthermore, two different glycosylated proteins, OsSERK1 and OsSERK2, were demonstrated to interact with OsMLD1. Overexpression of OsSERK1 and OsSERK2 in the protoplast of the *mld1* mutant could induce more severe cell death than in the WT. Furthermore, co-expression with *OsMLD1* could suppress OsSERK1- or OsSERK2-induced cell death in tobacco cells. Therefore, the *OsMLD1* may be involved in the control of glycosylated proteins by interacting with OsRpn1, and it is an important regulator of ER-stress induced cell death.

## 2. Results

### 2.1. The mld1 Mutant Exhibits Lesion Mimic Phenotypes Accompanied by Enhanced Disease Resistance

**Figure 1:**
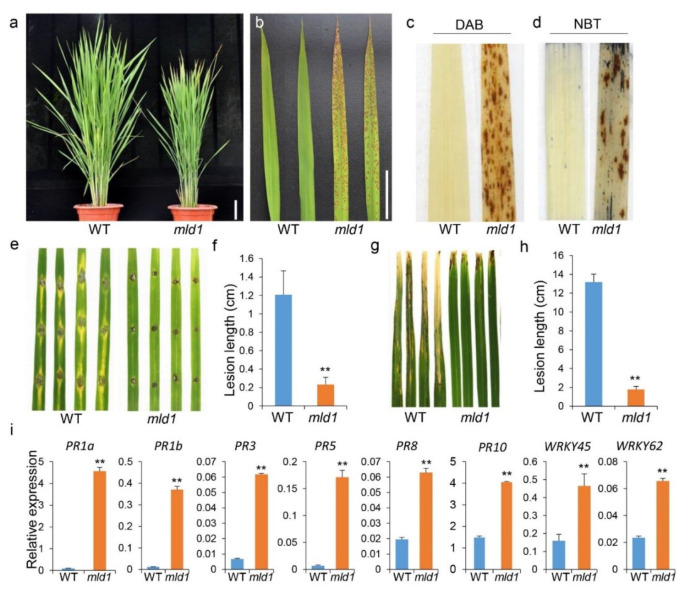
Phenotypic analysis of the *mld1* mutant of rice (*Oryza sativa*). (**a**,**b**) Plants at 90 days after seed sowing in the paddy field. Scale bar: 10 cm. (**c**,**d**) DAB (**c**) and NBT (**d**) staining of the WT and *mld1* mutant leaves to indicate the accumulation of H_2_O_2_ and O^2−^, respectively. (**e**,**f**) Leaves of the wildtype (WT) and *mld1* mutant plants inoculated with the compatible *M. oryzae* isolate H535. Phenotype and lesion length at 120 h after punch inoculation with H535 spores. Data are the means ± SD (*n* = 12). (**g**,**h**) Inoculation of the *mld1* mutant and WT plants with the *Xoo* isolate *PXO*99. Phenotype and lesion length at 14 dpi. Data represent the means ± SD (*n* = 4). (**i**) Expression analysis of several defense-related genes in the WT and *mld1* mutant plants based on RT-qPCR. Total RNA was extracted from the leaves of 30 day old seedlings. Data were normalized to the expression of the *OsACTIN1* gene. Values are the means ± SD (*n* = 3). Significance analysis of the data (**f**,**h**,**i**) was performed using Student’s *t*-test (** *p* < 0.01).

The *mld1* mutant with spontaneous spotted leaf was identified from the T-DNA inserted Geng (japonica) rice cv. Aichiasahi lines. Under normal field growth conditions (long day), the mutant *mld1* leaves were not significantly different from those of the WT before the booting stage. By contrast, *mld1* initially exhibited brown necrotic spots on the leaf apex of the top second leaves approximately 90 days post sowing, with these HR-like lesions becoming much more severe and gradually spreading throughout the leaves (Figure 1a,b). PCD is usually accompanied by the accumulation of intracellular reactive oxygen species (ROS) [13,22,56,57,58,59]. To identify the possible biochemical mechanisms underlying the development of the lesion mimic in *mld1*, we investigated the expression of histochemical markers for H_2_O_2_ and cell death. After 3,3′-diaminobenzidine (DAB) staining, the *mld1* mutant was stained with brown polymer deposition, while the WT leaf had no brown spots (Figure 1c), indicating that the accumulation of a large amount of H_2_O_2_ had occurred in the *mld1* leaves. Additionally, similar results were obtained when nitro blue tetrazolium (NBT) staining was used as an indicator of O^2−^ accumulation (Figure 1d). Most rice lesion mimic mutants confer enhanced resistance to pathogens [18,22,58,59]. To evaluate the disease resistance of *mld1*, the detached leaves of 1 month old plants of the WT and *mld1* mutant were punch-inoculated with *Magnaporthe oryzae* isolate H535. At 5 days post inoculation, the disease lesions on the *mld1* leaves were far less common than on the WT (Figure 1e,f). When the 2 month old plants were inoculated with the *Xanthomonas oryzae* pv. *oryzae* isolate PXO99, the lesions on the *mld1* leaves were much shorter than those on the WT plants at 2 weeks post inoculation (Figure 1g,h). These results indicated that the *mld1* mutant exhibited significantly enhanced resistance to both *M. oryzae* and *Xoo*. Consistent with enhanced disease resistance, RT-qPCR analysis showed that the expression levels of eight pathogenesis-related or defense-related genes, namely, *OsPR1a*, *OsPR1b*, *OsPR3*, *OsPR5*, *OsPR8*, *OsPR10*, *OsWRKY45*, and *OsWRKY62,* were significantly elevated in *mld1* (Figure 1i). These results suggest that *mld1* confers enhanced disease resistance accompanied with the activation of disease-resistant signaling pathways. Additionally, the *mld1* plants showed evident retardation in several agronomic traits compared with the WT plants, including reduced plant height, increased grain length, and a lower 1000-grain weight (Figure S1). The *mld1* plants also showed accelerated senescence accompanied by a reduced chlorophyll content in the flag leaves, as indicated by the SPAD value and upregulation of several senescence-associated genes (Figure S2).

### 2.2. Disruption of *OsMLD1* Leads to the Phenotypes of the mld1 Mutant

Genetic analysis showed that the *mld1* mutation was conferred by a recessive allele and was tightly linked to the T-DNA insertion (Table S1). Next, we performed SiteFinding TAIL-PCR to clone the sequence flanking the T-DNA. BLAST analysis revealed that the T-DNA was integrated into chromosome 3, which was further confirmed by specific PCR using primer sets flanking the insertion site and/or T-DNA borders. According to the prediction from the Rice Genome Annotation Project (http://rice.plantbiology.msu.edu/, accessed on 25 September 2018), the insertion site is located 2672 bp downstream of the translation termination site of candidate gene 1 (*G1*, *LOC_Os03g03290*) and 1347 bp downstream of the translation termination site of candidate gene 2 (*G2*, *LOC_Os03g03300*) (Figure S3a,b). To determine whether the T-DNA insertion caused any change in the expression level of both candidate genes, semiquantitative RT-PCR and RT-qPCR analyses showed that the expression level of *G1* in *mld1* was dramatically lower than that in the WT, while the expression of *G2* demonstrated no detectable difference between the WT and *mld1* (Figure S3c,d), suggesting that the *mld1* mutation was associated with the disruption of *G1*, which was renamed *OsMLD1*. RT-qPCR analysis showed that the *OsMLD1* was expressed in all organs at different developmental stages, with the strongest expression detected in the panicles (Figure S3e). To determine whether the downregulation of *OsMLD1* was indeed associated with the *mld1* mutant phenotype, we generated knockout and overexpression plants of *OsMLD1* (Figure S4). The knockout plants (*KN*) exhibited cell death that was even more severe than that of *mld1*, while the overexpression plants (*OE*) had the same phenotype as the WT (Figure S4g). The *KN* plants also showed enhanced resistance to *M. oryzae* and elevated expression of pathogenesis-related genes (Figure S5). These results confirmed that the mutation of *OsMILD1* led to the phenotypes in the *mld1* mutant.

### 2.3. *OsMLD1* Encodes a Malectin That Is Associated with the ER and Golgi under Normal Conditions

**Figure 2:**
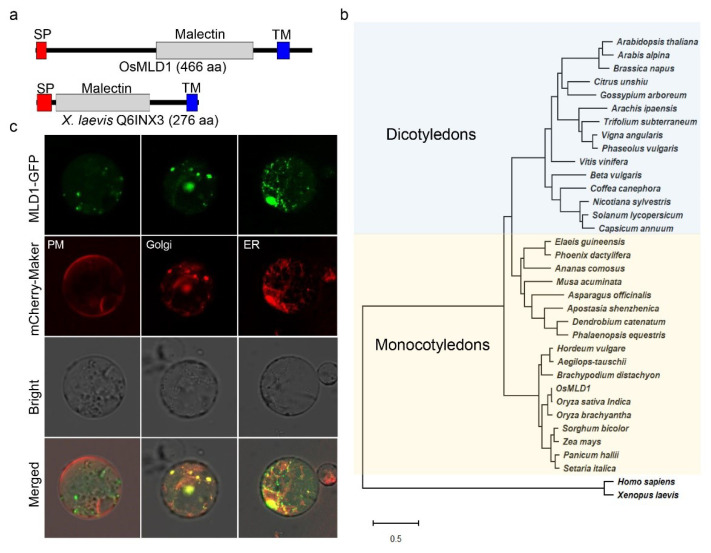
OsMLD1 is an ER- and Golgi-associated protein. (**a**) Schematic representation of *OsMLD1* and its homolog from *Xenopus laevis* (Q6INX3). SP indicates signal peptide; TM indicates the transmembrane domain. (**b**) Phylogenetic tree of *OsMLD1* and its homologs from other plant species. *Oryza sativa* Geng, OsMLD1; *Oryza sativa* Indica, EAY88339.1; *Oryza brachyantha*, XP_006650992.1; *Sorghum bicolor*, XP_002468603.1; *Panicum hallii*, PAN51864.1; *Setaria italic*, XP_004985905.1; *Hordeum vulgare* subsp. *Vulgare*, BAJ97982.1; *Zea mays*, NP_001151098.1; *Brachypodium distachyon*, XP_003558932.1; *Aegilops tauschii* subsp. *Tauschii*, XP_020150979.1; *Elaeis guineensis*, XP_010933987.1; *Ananas comosus*, XP_020093405.1; *Phoenix dactylifera*, XP_008807843.1; *Musa acuminata* subsp. *Malaccensis*, XP_009382243.1; *Arabidopsis thaliana*, AT4G00300; *Apostasia shenzhenica*, PKA48091.1; *Dendrobium catenatum*, XP_020686577.1; *Phalaenopsis equestris*, XP_020597844.1; *Asparagus officinalis*, XP_020261801.1; *Arabis alpine*, KFK30615.1; *Solanum lycopersicum*, XP_004229933.1; *Beta vulgaris* subsp. *Vulgaris*, XP_010669972.1; *Brassica napus*, XP_013732165.1; *Citrus unshiu*, GAY51467.1; *Vitis vinifera*, XP_002278060.1; *Vigna angularis*, XP_017418921.1; *Coffea canephora*, CDP08774.1; *Nicotiana sylvestris*, XP_009757788.1; *Capsicum annuum*, XP_016538110.1; *Arachis ipaensis*, XP_016193902.1; *Trifolium subterraneum*, GAU28640.1; *Phaseolus vulgaris*, XP_007139802.1; *Gossypium arboretum*, XP_017637458.1; *Homo sapiens*, NP_055545.1; *Xenopus laevis*, Q6INX3. The phylogenetic tree was created using the maximum likelihood method in MEGA X software. Bootstrap support values from 1000 pseudo-replicates of the dataset are provided as percentages at the corresponding nodes when >50%. (**c**) Subcellular localization of MLD1 in rice protoplasts. Co-expression of MLD1-GFP and mChery-HDEL (ER), Man1-mChery (Golgi), and PIP2A-mChery (PM) in the rice protoplasts.

*OsMLD1* encodes a protein that is 466 amino-acid residues in length. Prediction with SMART (http://smart.embl-heidelberg.de/, accessed on 19 October 2018) and InterPro (https://www.ebi.ac.uk/interpro/, accessed on 19 October 2018) showed that *OsMLD1* consists of a signal peptide (SP) at the *N*-terminus, a malectin domain at the center, and a transmembrane domain (TM) at the *C*-terminus. The structure of the soluble domain of *OsMLD1* resembles that of ANX1/2 and FER, which was already shown to feature a tandem malectin domain, as described in detail in [60,61,62], whereas it differs from that of the first identified malectin from *Xenopus laevis* (accession No. Q6INX3), despite sharing weak conservation of residues (Figure 2a and Figure S3f). In plants, a subfamily of receptor-like kinase proteins identified from several species contain a malectin domain at the *N*-terminus, playing roles in plant defense response activation [63], while few proteins containing only a malectin domain have been characterized. A search against NCBI using the *OsMLD1* sequence as a query identified 34 homologs from different plant species. Phylogenetic analysis showed that *OsMLD1* is more closely related to the homologs of monocot plants (Figure 2b), implying similar biological functions. In animals, malectin is mainly localized in the ER under normal conditions [46,64]. To determine the subcellular localization of OsMLD1, MLD1-GFP was transiently co-expressed with mCherry-HDEL, mCherry-Golgi, and mCherry-PM in rice protoplasts. The GFP signals were overlaid with mCherry in the ER, as well as in the Golgi, but not in PM (Figure 2c), suggesting that *OsMLD1* is an ER- and Golgi-associated protein. In addition, the above 34 *OsMLD1* homologs from different plant spices were also predicted as ER membrane resident proteins, suggesting their similar functions.

### 2.4. Disruption of *OsMLD1* Induces Prolonged ER Stress

**Figure 3:**
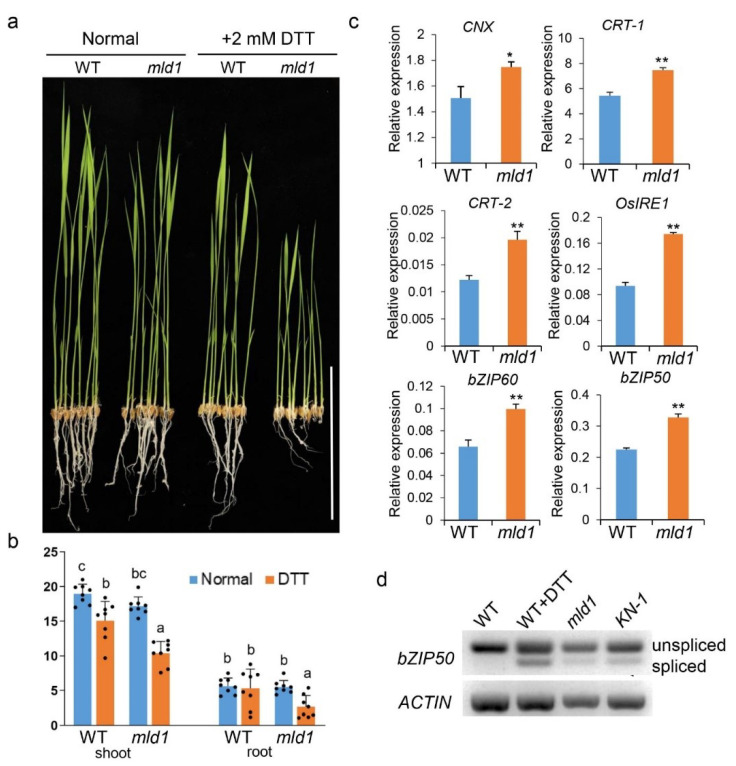
*mld1* is more sensitive to ER stress. (**a**) The growth of the WT and *mld1* mutant in liquid medium with 2 mM DTT refreshed daily was observed over a period of 7 days. Liquid medium without DTT served as a control. (**b**) Quantitative measurements of the lengths of the shoots and roots of wildtype and *mld1* seedlings in three trials. Values are the means ± SD (*n* = 8). Significance analysis was performed using two-factor ANOVA with Tukey’s HSD test. Different letters indicate significant differences (*p* < 0.05). (**c**) ER stress-induced expression of several genes. mRNA levels of representative ER stress-induced genes in the WT and *mld1* seedlings. RT-qPCR analyses of three biological replicates. Data were normalized to the expression of the *OsACTIN1* gene. Values are the means ± SD (*n* = 3), Significance analysis of the data was performed using Student’s *t*-test (* *p* < 0.05, ** *p* < 0.01). (**d**) ER stress-induced splicing of *OsbZIP50* mRNA in *mld1*. Unspliced (U) and spliced (S) *OsbZIP50* forms co-exist in *mld1*. The WT treated with DTT was used as a positive control.

Many studies have revealed that prolonged or unresolved ER stress causes PCD [65,66]. Given that the *mld1* mutant exhibits a lesion mimic phenotype and *OsMILD1* is an ER-localized protein, we reasoned that the disruption of *OsMLD1* would induce prolonged ER stress, resulting in PCD. To investigate whether the PCD that occurred in *mld1* was related to the disruption of ER stress signaling, we first tested the response of *mld1* seedlings to dithiothreitol (DTT), an ER stress stimulus. The result showed that the growth of *mld1* seedlings under 2 mM DTT treatment was severely retarded compared to the WT (Figure 3a,b), suggesting that *mld1* was more sensitive to ER stress compared to the WT. Secondly, we conducted RT-qPCR analysis and found that the expression levels of several UPR target genes [67], including *CNX*, *CRT-1*, *CRT-2*, *bZP60*, *bZIP39, bZIP28, NEF*, *ERdj3-like*, *OsVPE2*, and *OsVPE3* were significantly upregulated in the *mld1* seedlings (Figure 3c and Figure S6). Notably, the expression level of *IRE1*, which functions as an ER stress sensor, was also upregulated in *mld1*, further indicating that *OsMLD1* is involved in ER stress sensing. In *Arabidopsis* and rice, the activation of IRE1 by ER stress leads to the nonconventional cytoplasmic splicing of *AtbZIP60* [68,69] and *OsbZIP50* [67], resulting in the production of bZIP transcription factors that enter the nucleus and activate UPR target genes. Consistent with the upregulation of *IRE1*, the expression level of *bZIP50* was significantly upregulated in the *mld1* seedlings (Figure 3c). The form of spliced *bZIP50* was detected in the leaves of *mld1* and the CRISPR/Cas9-mutated line *KN-1* under normal conditions, as well as in the WT under treatment with DTT, whereas only unspliced transcripts of *bZIP50* could be detected in the WT under normal conditions (Figure 3d). These results demonstrated that the disruption of *OsMLD1* induced IRE1-mediated ER stress and activated UPR signaling in the *mld1*.

### 2.5. OsRpn1 Is an ER Localized Protein and Interacts with OsMLD1

**Figure 4:**
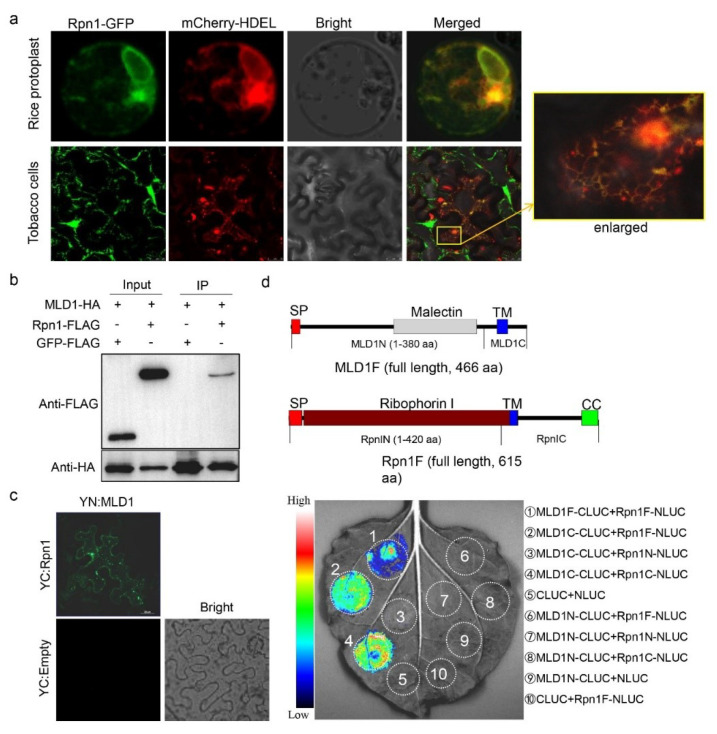
Rice ribophorin I (OsRpn1) localizes in the ER and interacts with OsMLD1. (**a**) Subcellular localization of OsRpn1 in rice protoplasts and *Nicotiana benthamiana* leaves. (**b**) Co-IP assays for the interactions of *OsMLD1* with OsRpn1 *in vivo*. Rpn1-GFP (~110 kD), MLD1-FLAG (~50 kD), and GUS-FLAG (~75 kD) were expressed in *Nicotiana benthamiana.* Co-IP was performed using GFP-binding beads. Proteins were detected with antibodies as indicated. Similar results were obtained in three independent experiments. (**c**) Interactions between *OsMLD1* and OsRpn1 shown by BiFC assays in *N. benthamiana* leaf epidermal cells. BiFC fluorescence is indicated by the eYFP signal. The eYFP signal was not detected in the corresponding negative controls. (**d**) Firefly LUC complementation imaging (LCI) assay detecting the interaction between *OsMLD1* and OsRpn1. The colored scale bar indicates the luminescence intensity. NLUC indicates the N-terminus of LUC, while CLUC indicates the C-terminus of LUC.

In animals, malectin interacts with ribophorin I and participates in ER quality control for glycoproteins [70,71]. Considering that the *mld1* mutant displayed phenotypes similar to those under ER stress, we assumed that *OsMLD1* interacts with the ribophorin I homolog in rice. Using the amino-acid sequence of *Homo sapiens* ribophorin I (accession No. AAH10839) as a query, we searched the rice genome and then cloned the highest homolog of the ribophorin I gene from the rice cultivar ‘Aichiasahi’ (OsRpn1, *LOC_Os05g23600*). Rice OsRpn1 encodes a protein that is 615 aa long, which shares 31.66% amino-acid identity to *Homo sapiens* RpnI (Figure S7a). Phylogenetic analysis showed that OsRpn1 is conserved in numerous plant species, but is more closely related to the homologs of monocot plants (Figure S7b). Similar to *Homo sapiens* RpnI, OsRpn1 consists of a signal peptide in the *N*-terminus, a ribophorin I domain overlaid with a transmembrane region at the center, and a coiled-coli domain in the *C*-terminus (Figure 4c and Figure S7a). When the OsRpn1-GFP was transiently co-expressed with mCherry-HDEL in rice protoplasts and the epidermal cells of *N. benthamiana*, the GFP signals were overlaid with mCherry in the ER (Figure 4a), indicating that OsRpn1 is an ER-localized protein. To confirm the hypothesis that *OsMLD1* interacts with OsRpn1, a co-immunoprecipitation (Co-IP) assay was performed. We transiently co-expressed OsRpn1-Flag or GFP-Flag (as the negative control) with OsMLD1-HA in rice protoplast and pulled down HA-tagged proteins with anti-HA beads. The OsRpn1-Flag, but not the GFP-Flag, was successfully co-immunoprecipitated with OsMLD1-HA, indicating that *OsMLD1* interacts with OsRpn1 (Figure 4b). We also transiently co-expressed OsRpn1-GFP with OsMLD1-Flag or GUS-Flag (as the negative control) in *N. benthamiana* and pulled down GFP-tagged proteins with anti-GFP beads. Only the MLD1-Flag was successfully co-immunoprecipitated with Rpn1-GFP, further indicating that *OsMLD1* interacts with OsRpn1 (Figure S8). A bimolecular fluorescence complementation (BiFC) assay [72] was used to further confirm the interaction in planta. *N. benthamiana* epidermal cells co-expressing MLD1-eYFP^C^ and OsRpn1-eYFP^N^ showed clear YFP fluorescence. In contrast, no fluorescence was observed when YN:MLD1 was co-transformed with the YC moiety alone (Figure 4c). Furthermore, a firefly luciferase complementation imaging (LCI) system was used to validate the interaction. Using a low-light imaging system, fluorescence signals were detected when MLD1F-CLUC and Rpn1F-NLUC, MLD1C-CLUC and Rpn1F-NLUC, or MLD1C-CLUC and Rpn1C-NLUC were co-expressed in *N. benthamiana* leaves. No fluorescence signals were detected when MLD1N-CLUC was co-transformed with Rpn1F-NLUC, Rpn1N-NLUC, or Rpn1C-NLUC. Fluorescence signals were also not detected when MLD1C-CLUC was co-transformed with Rpn1N-NLUC, as well as the three negative controls (Figure 4d). These results demonstrate that *OsMLD1* interacts with OsRpn1, and the *C*-termini of both proteins are required for this interaction.

### 2.6. Disruption of *OsMLD1* Affects the N-Glycosylation Process

**Figure 5:**
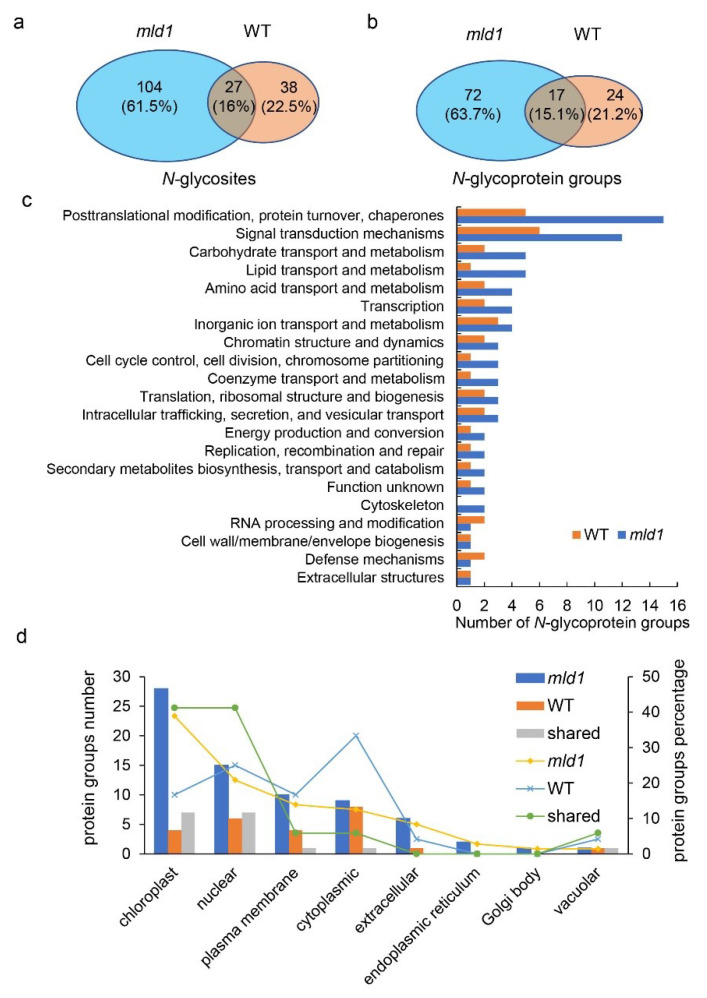
*N*-Glycoproteome analysis of the wildtype and *mld1* mutant. (**a**,**b**) Overlap of the *N*-glycosites and *N*-glycoprotein groups from *mld1* and the WT. (**c**) Distribution of *N*-glycoproteins based on COGs. (**d**) Subcellular location of protein groups.

To determine the difference in the *N*-glycosylation process in the *mld1* mutant and WT, we used a proteomics method based on ConA lectin affinity chromatography enrichment and high-resolution liquid chromatography–tandem mass spectrometry (LC-MS/MS) [73,74]. We obtained 131 and 65 *N*-glycosites in total, corresponding to 89 and 41 *N*-glycoprotein groups in the seedlings of *mld1* and WT, respectively (Figure 5a,b; Table S2). The peptide length ranged between seven and 41 amino-acid residues (Table S3), consistent with the expected tryptic profile. *N*-Glycosylated motif enrichment analysis revealed 42.3% N–X–S, 32.4% N–X–T, and 25.6% N–X–X–N (where X represents any amino acid except proline) (Table S4). The glycoproteomics analysis showed there were about three times the number of identified *N*-glycosites and *N*-glycoprotein groups from *mld1* compared to the WT, implying a major difference in *N*-glycosylation in the *mld1* mutant and the WT. The *N*-glycosite and *N*-glycoprotein group overlaps were 16% and 15.1%, respectively (Figure 5a,b). To further investigate the associated biological processes on the basis of the differentially expressed *N*-glycoproteins, all identified *N*-glycoprotein groups were subjected to analysis using COGs (Clusters of Orthologous Groups). For biological process, the identified *N*-glycoproteins from both *mld1* and the WT were associated with some metabolic processes, consistent with a previous study [74]. The major differences in *N*-glycoproteins between *mld1* and the WT were also mainly associated with ‘post-translational modification, protein turnover, chaperones, and signal transduction mechanisms’ (Figure 5c; Table S5). Subcellular location analysis showed that the different glycoproteins were mainly located in the chloroplast, plasma membrane, extracellular space, and the nucleus (Figure 5d; Table S6). Taken together, our data suggest that the disruption of *OsMLD1* obviously affects the *N*-glycosylation process, implying that *OsMLD1* may play an essential role in backup glycoprotein quality control.

### 2.7. *OsMLD1* Interacts with OsSERK1 or OsSERK2 and Suppresses OsSERK1- or OsSERK2-Induced Cell Death

**Figure 6:**
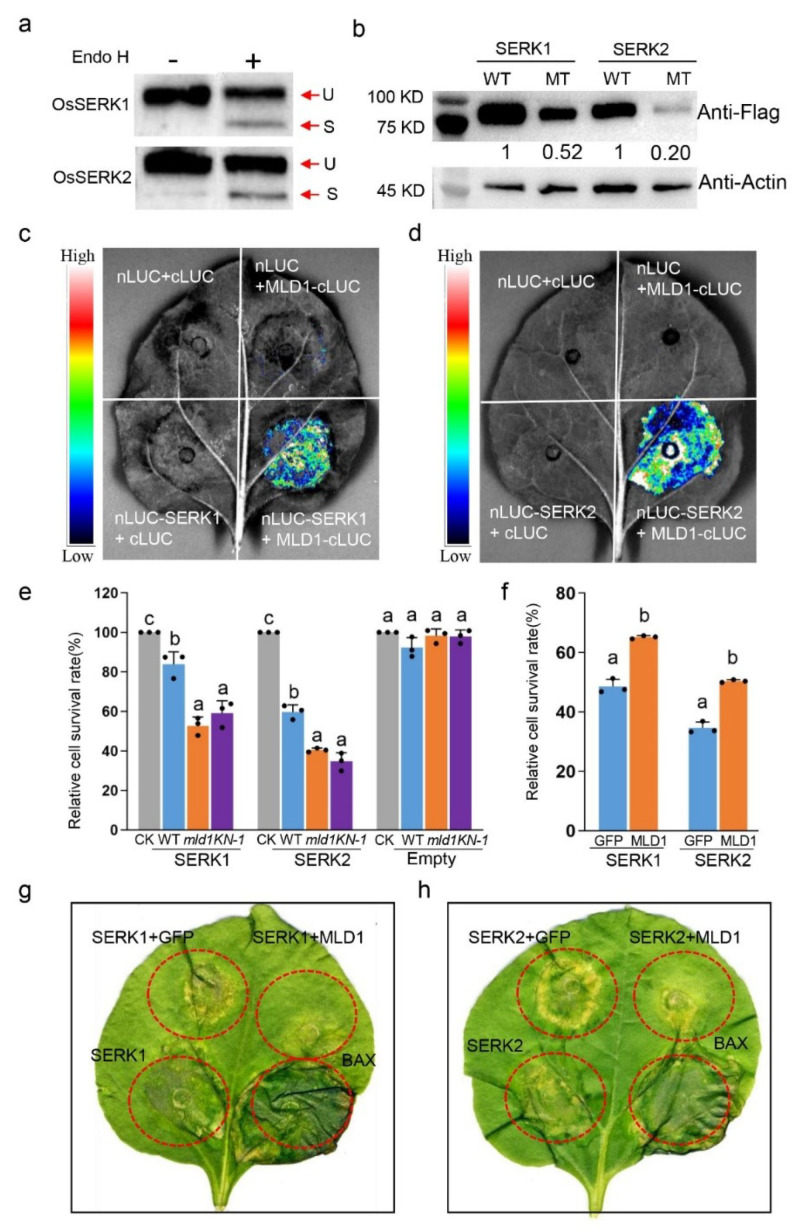
OsMLD1 interacts with OsSERK1 and OsSERK2 and suppresses their induced cell death. (**a**) Western blot analysis with anti-FLAG showed that glycans were cleaved from OsSERK1 or OsSERK2 by Endo H. Red arrow indicates uncleaved (U) or cleaved. (**b**) Western blot analysis OsSERK1 or OsSERK2 transiently expressed in protoplast of wildtype and *mld1* mutant. (**c**,**d**) Firefly LUC complementation imaging (LCI) assay detecting the interactions of *OsMLD1* with OsSERK1 and OsSERK2, respectively. (**e**) Survival rates of protoplasts measured in WT, *mld1,* and *KN-1.* OsSERK1, OsSERK2, the empty vector, and luciferase were co-expressed in the protoplasts of the WT, *mld1* and *KN-1*, respectively. The only pUBI:LUC plasmid DNA was used to normalize the protoplasts in each treatment, representing 100% viable cells. In contrast, survival rates after OsSERK1 and OsSERK2 transformation were obtained. The empty vector was used as a negative control in each treatment. Data are the means ± SD (*n* = 3). Significance analysis was performed using two-factor ANOVA with Tukey’s HSD test. Different letters indicate significant differences (*p* < 0.05). (**f**) Survival rate of co-expressed OsSERK1 or OsSERK2 with OsMLD1-GFP or GFP in *mld1* protoplasts. (**g**,**h**) *OsMLD1* suppresses OsSERK1- or OsSERK2-induced cell death in *N. benthamiana*. OsSERK1 and OsSERK2 alone or with *OsMLD1* were transformed into *N. benthamiana* epidemical cells. The co-expression of OsSERK1 with GFP and OsSERK2 with GFP was used as a negative control, and BAX was used as the positive control.

Among the different glycosylated proteins revealed by *N*-glycoproteome analysis, two characterized proteins, OsSERK1 and OsSERK2, showed high detectable glycosylation intensity in *mld1* but not in the WT (Table S2). We speculated that the glycosylation quality control of the two proteins may be disturbed in *mld1*; thus, the functions of the two proteins may be affected. To verify this point, firstly, the *C*-terminal FLAG-tagged OsSERK1 or OsSERK2 was transiently transformed into rice protoplast. Crude proteins were extracted and digested with proteinase Endo H. Western blot analysis with anti-FLAG showed that the glycans were cleaved from both OsERRK1 and OsSERK2 by Endo H (Figure 6a). Consistently, FLAG-tagged OsSERK1 or OsSERK2 transiently expressed in *N. benthamiana* epidermal cells was also cleaved by Endo H and PNGase F (Figure S9), indicating that both proteins were glycosylated proteins. Secondly, GFP-tagged OsSERK1 or OsSERK2 was transiently co-expressed with mCherry-PM in rice protoplasts. The GFP signals were overlaid with mCherry in the PM (Figure S10), suggesting that both OsSERK1 and OsSERK2 are PM-associated proteins. When Flag-tagged OsSERK1 or OsSERK2 was transiently expressed in protoplast of the WT and *mld1* mutant, Western blot analysis showed that amounts of both OsSERK1 and OsSERK2 were significantly reduced in *mld1* mutant (Figure 6b), suggesting that disruption of *OsMLD1* affected the expression of OsSERK1 and OsSERK2. Thirdly, LCI assays were used to determine the interaction between *OsMLD1* and OsSERK1 or OsSERK2. Fluorescence signals were detected when MLD1F-CLUC and NLUC-SERK1 or OsMLD1C-CLUC and NLUC-SERK2 were co-expressed in *N. benthamiana* leaves (Figure 6c,d). These results indicated that *OsMLD1* interacted with OsSERK1 and OsSERK2 in planta. Furthermore, OsSERK1 or OsSERK2 and an empty vector were co-expressed with luciferase in the protoplasts of the WT, *mld1,* and *KN-1* lines. Living cells were measured by fluorescence intensity. In each assay, the protoplasts transformed with only luciferase was used as an indicator of surviving cells. The results showed that both OsSERK1 and OsSERK2 induced cell death in three backgrounds, but cell death in the *mld1* mutant and *KN-1* line was more severe than that in the WT (Figure 6e). In addition, OsSERK1 or OsSERK2 was co-expressed with GFP only or OsMLD1-GFP in the protoplasts of the *mld1*, while the luciferase was also co-expressed to measure the live cells by fluorescence intensity. The survival rate of protoplasts co-expressing OsSERK1 or OsSERK2 with OsMLD1-GFP was significantly higher than that of those co-expressing with GFP only (Figure 6f). These results suggested that the disfunction of *OsMLD1* led to a defect in suppressing OsSERK1- or OsSERK2-induced cell death. Consistently, transient overexpression of OsSERK1 or OsSERK2 alone in *N. benthamiana* leaves could induce cell death, as well as BAX. This cell death could be suppressed by co-expression with OsMLD1-GFP, but not with GFP (Figure 6g,h), further indicating that *OsMLD1* is involved in suppressing OsSERK1- and OsSERK2-induced cell death.

## 3. Discussion

### 3.1. Rice mld1 Represents a Novel Type of LMM That Results from the Disruption of the Malectin Gene

PCD, as a fundamental biological cellular process in eukaryotes, controls cell suicide in a conserved and genetically regulated manner, causing the death of single cell, specific tissues, or whole organs [75,76,77]. The hypersensitive response (HR) is considered a specialized form of PCD and is a defense mechanism that is triggered by pathogens and insects [78]. Extensive insight into the understanding of plant PCD in response to stress originated from the identification of many LMMs that display spontaneous HR-like cell death. LMMs constitute a valuable genetic resource for deciphering the PCD pathway and plant immunity. To date, more than 30 LMM genes have been characterized from rice. These genes encode a wide range of proteins, suggesting that PCD and plant immunity are complex biological processes that encompass many signaling pathways. In this study, we characterized a T-DNA insertion mutant LMM *mld1*. Under normal field conditions, spontaneous HR-like lesions occurred on the leaf apex of the top second leaves approximately 90 days post sowing, with these HR-like lesions becoming more severe and gradually spreading throughout the leaves. Similar to most characterized LMMs, the *mld1* mutant also showed broad-spectrum resistance against fungal and bacterial pathogens, which was accompanied by the upregulation of defense-related genes and ROS burst (Figure 1). In the *mld1* mutant, the T-DNA insertion site was located downstream of the translation termination site of the gene *OsMLD1*, which encodes a malectin protein. The T-DNA insertion led to the significant downregulation of *OsMLD1*, resulting in defense-related phenotypes. This was supported by the CRISPR/cas9-mutated lines (*KN*) of *OsMLD1*, which showed lesion mimic phenotypes and enhanced disease resistance compared to the WT (Figures S4 and S5). Therefore, the *mld1* mutant represents a novel LMM that results from the disruption of a malectin gene. Notably, the *OsMLD1*-*KN* lines exhibited lesion mimic phenotypes 30 days earlier than the *mld1*, and the necrosis of the *KN* lines was more severe than that of the *mld1*. This may be due to the precise regulation of *OsMLD1* expression, providing a clue for manipulating its expression to balance cell death and growth. The *mld1* mutant was more sensitive to DTT treatment, accompanied by activation of ER stress sensor gene *OsIRE1* and several UPR target genes. Consistent with activation of *OsIRE1* and *OsbZIP50*, the *mld1* and its CRISPR/cas9-mutated line produced spliced mRNA of normal conditions, similar to WT plants treated with DTT (Figure 3). These results suggest that the cell death resulting from disruption of *OsMLD1* is ER stress-induced, which is mediated by the IRE1–bZIP60 pathway. In addition, the rice genome likely contains only one copy of the *OsMLD1* gene, and a large number of plant species have *OsMLD1* homologs, implying that malectin may play conserved and important roles in the plant kingdom.

### 3.2. *OsMLD1* Interacts with Ribophorin I, a Unit of the OST Complex, and Participates in Protein N-Glycosylation Modification

Malectin was first identified in *X. laevis*, but it is also present in other animals and selectively binds carbohydrates for diglucose and high-mannose *N*-glycans [45]. Under normal conditions, malectin is an ER membrane-localized protein that preferentially associates with misfolded glycoproteins and inhibits their secretion [79]. Malectin forms a stable complex with an ER-resident trans-membrane protein, ribophorin I. The co-expression of malectin and ribophorin I significantly enhanced the association between malectin and a folding-defective protein and reduced its secretion; however, the secretion of WT proteins was not affected [70]. Ribophorin I is a core unit of the OST complex and acts as a substrate-specific facilitator of *N*-glycosylation, regulating substrate delivery to the OST core [48,80]. Suppression of ribophorin I leads to the movement of malectin from the ER to the Golgi, indicating that the subcellular localization of malectin is closely regulated by the expression level of ribophorin I [64]. Mammalian malectin participates in ER quality control for glycoproteins [46,71]. So far, only a few malectins from plants have been characterized. Here, we demonstrated that rice malectin *OsMLD1* is structurally similar to animal malectin and is conserved in many plant species (Figure 2a,b). *OsMLD1* is an ER- and Golgi-associated protein in rice protoplasts and tobacco epidermal cells (Figure 2c). The different subcellular localizations between *OsMLD1* and its animal counterparts, which are mainly localized in the ER under normal conditions, may contribute to their different biological functions. Similar to animal malectins, *OsMLD1* interacts with the ribophorin I homolog OsRpn1 in planta, and the C-termini of both *OsMLD1* and OsRpn1 are required for this interaction (Figure 4). We reasoned that this interaction may account for the association with unfolded and/or misfolded proteins to maintain a high level of quality control. This point was supported, at least partially, by the *mld1* mutant, which showed greater sensitivity to DTT treatment, while several UPR genes were constitutively upregulated (Figure 3 and Figure S6). Furthermore, using ConA lectin affinity chromatography enrichment and high-resolution LC–MS/MS analysis, we found there are many much *N*-glycosites and *N*-glycoproteins in *mld1* than in WT (Figure 5), suggesting that the *N*-glycosylation process was affected by disruption of *OsMLD1*. These data suggested that the plant malectin, which possibly plays similar roles to its animal homologs, participates in protein *N*-glycosylation modification and ER quality control.

### 3.3. OsMLD1-Mediated Protein N-Glycosylation Modification Is Essential for OsSERK1- and OsSERK2-Induced Cell Death

SERKs (somatic embryogenesis receptor-like kinases) are a small group of leucine-rich repeat RLKs that play critical roles in the early events of brassinosteroid (BR) signal transduction [81]. In *Arabidopsis*, there are five SERK homologs: SERK1–SERK5 [82]. Previous studies demonstrated that the SERK family members SERK3 (also named BAK1) and SERK4 function in immunity by associating with the immune receptors FLS2, EFR, and PEPR1/2 [83]. In addition, BAK1 and SERK4 negatively regulate the plant cell death process [84,85]. BAK1/SERK4-regulated cell death is suppressed by the mutation of STT3a, a protein involved in *N*-glycosylation modification, suggesting that *N*-glycosylation and specific ERQC components are essential for activating *bak1/serk4* cell death. In addition, a cysteine-rich receptor-like kinase is likely among the client proteins of protein glycosylation involved in BAK1/SERK4-regulated cell death [86]. SERK1 is also involved in immune signaling in transgenic plants expressing the tomato Ve1, a receptor like protein [87]. In rice, several studies suggested that rice SERK proteins are involved in the rice immune response. Nonspecific silencing of the two rice SERK genes and several closely related genes in rice compromises resistance against the fungal pathogen *M. oryzae* [88]. Silencing of *OsSERK2* compromises Xa21-mediated immunity to *Xoo*, and OsSERK2 is also involved in Xa3- and FLS2-mediated immunity [83]. Conversely, the overexpression of *OsSERK2* (referred to as OsSERK1 in the original publication) enhances resistance against *M. oryzae* [89]. In this study, we found great differences in *N*-glycosylation between the WT and *mld1* mutant. Among the differential *N*-glycosylated peptides, one represents two of the rice SERK proteins: OsSERK1 and OsSERK2 (Table S2). Further investigation demonstrated that *OsMLD1* interacts with both OsSERK1 and OsSERK2 (Figure 6c,d). Transient overexpression of OsSERK1 or OsSERK2 induced cell death in both the WT and the *mld1* mutant protoplasts, and the cell death in *mld1* was more severe than that in the WT; furthermore, co-expression with *OsMLD1* could reduce OsSERK1- or OsSERK2-induced cell death in *mld1* protoplasts (Figure 6e,f). Considering that OsSERK1 and OsSERK2 had higher glycosylation intensity in *mld1* than in the WT, and amounts of OsSERK1 and OsSERK2 were reduced in the *mld1* mutant, we hypothesized that the disruption of *OsMLD1* resulted in the accumulation of mis-glycosylation-modified OsSERK1 and OsSERK2, which induced cell death. Consistent with this hypothesis, the transient overexpression OsSERK1 or OsSERK2 induced the cell death of tobacco epidemical cells, and this death could be suppressed by the co-expression of *OsMLD1* (Figure 6g,h). These data inferred that OsMLD1-mediated protein *N*-glycosylation modification is essential for OsSERK1- or OsSERK2-induced cell death. In addition to OsSERK1 and OsSERK2, a dozen differential *N*-glycosylated proteins were annotated as immune receptor-like kinases, although many of them were not functionally characterized (Table S2), implying there are common mechanisms via which malectin-mediated protein *N*-glycosylation modification is essential for RLK (RLP)-mediated immunity. Consistently, a recent study showed that a malectin named SlRLK-like affects the localization and abundance of LeEIX2, another receptor-like protein in tomato, resulting in the suppression of EIX-induced immune responses [54].

### 3.4. A Proposed Working Model for OsMLD1

**Figure 7:**
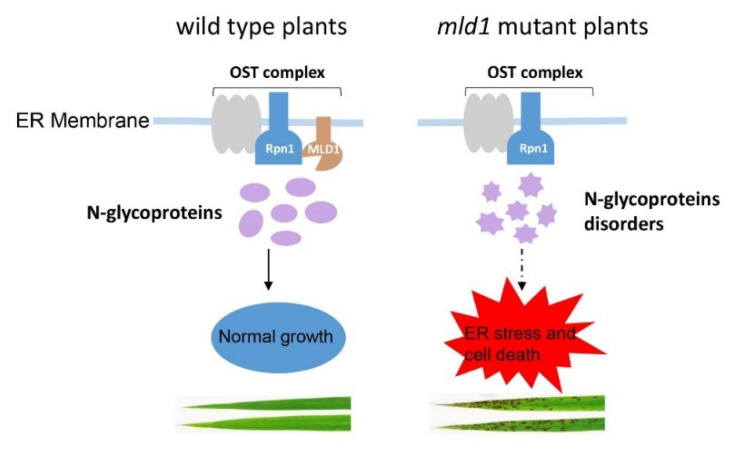
A proposed working model of the function of *OsMLD1* in ER quality control. In the wildtype, *OsMLD1* (malectin) forms a complex with ribophorin I (OsRpn1), which is one of the subunits of the OST complex. The interaction between *OsMLD1* and OsRpn1 may account for the normal process of *N*-glycoproteins. In the *mld1* mutant, the disruption of *OsMLD1* disturbs the association with *N*-glycoproteins, resulting in the normal modification of glycoproteins to be affected, which leads to prolonged ER stress in the *mld1* plants.

We propose a simple working model for *OsMLD1* (Figure 7), which provides new insight into understanding the crosstalk between *N*-glycosylation modification and ER stress-induced cell death. According to this model, in the WT, *OsMLD1* forms a complex with OsRpn1, a core unit of the OST complex, accounting for the protein’s *N*-glycosylation modification. In the *mld1* mutant, the deformation of the complex of *OsMLD1* and OsRpn1 results in perturbed *N*-glycosylation modification of a large number of proteins, including some RLKs (i.e., OsSERK1 and OsSERK2), leading to prolonged ER stress and, thus, contributing to cell death and immunity.

## 4. Materials and Methods

### 4.1. The Plant Materials and Growth Conditions

The rice lesion mimic mutant *mld1* was isolated from a T-DNA insertion library of *japonica* cv. *Aichiasahi* (wild-type, WT). All materials were conventionally grown in the experimental field of China Agricultural University (Beijing, China) in the standard rice-growing season. The phenotypic status of the lesion mimic was observed and recorded during the growth stage. The agronomic traits were statistically analyzed on the basis of 10 individual plants randomly selected from the WT and *mld1* plants, including plant height, tiller number, and 1000-grain weight.

The SPAD values of the leaves were measured as described previously [7,22], with some modifications, from the day of flowering and then every five days thereafter until 30 days in 15 plant replicates. The degree of leaf greenness was measured using a Minolta Chlorophyll Meter SPAD-502 (Minolta Camera Co., Ltd., Osaka, Japan) and was designated as the SPAD value.

### 4.2. T-DNA Flanking Sequence Isolation

SiteFinding thermal asymmetric interlaced (TAIL)-PCR [90] was used to isolate regions flanking the T-DNA insertion in *mld1.* The products of SiteFinding TAIL-PCR were sequenced and searched against the rice genome database (http://rice.plantbiology.msu.edu/, accessed on 20 September 2018) to obtain the T-DNA insertion site. Specific primers, R3, L3, L13-1, and L13-2 were used to reconfirm the T-DNA insertion site. The primers and sequences are listed in Table S7.

### 4.3. RNA Isolation and RT-qPCR Analysis

Total RNA was extracted with an RNA Prep Pure Plant kit (Zomanbio, Beijing, China) following the manufacturer’s instructions. Each RNA sample (1 μg) was reverse-transcribed using the QuantiTect reverse transcription kit (Vazyme, Nanjing, China). The real-time quantitative PCR (RT-qPCR) was performed using an ABI 7500 system (Thermo Fisher Scientific, Waltham, MA, USA) with the SYBR Premix Ex Taq (Genestar, Beijing, China). Leaves of five-leaf-stage WT and *mld1* plants were used to detect the expression of *OsMLD1* and several defense marker genes. Two-leaf-stage WT seedling leaves, stems, and roots, as well as the adult flag leaves (1 L), top three leaves (2–4 L), young panicles, and stems, were used for tissue-specific expression analysis of *OsMLD1*, respectively. All of the experiments were performed with three technical replicates and three biological replicates. The *OsACTIN* gene in rice was used as an internal control. The sequences of all of the primers used in the study are listed in Table S7. Additionally, the primer information of some genes, including senescence-associated genes and PR genes, was obtained from previous studies [19,22].

### 4.4. Vector Construction and Rice Transformation

Overexpression vectors (*pUBI::3FLAG-OsMLD1*) were constructed by inserting the corresponding cDNA fragments from cv. ‘Aichiasahi’ into the pCAMBIA1301-UBI vector. *pUBI::3FLAG-OsMLD1* contained the full-length coding sequence (CDS) of *OsMLD1* (1401 bp). The knockout vector was constructed with the approaches described previously. A 20 bp gene-specific spacer sequence of *OsMLD1* was cloned into the vector pOs-sgRNA and then recombined into the CRISPR/Cas9-containing vector [91]. All the constructs were verified by sequencing and were subsequently introduced into rice cv. ‘Aichiasahi’ by *Agrobacterium tumefaciens*-mediated transformation as described previously [92]. The primers used for building the above constructs are listed in Table S7.

### 4.5. Histochemical Analysis

Fresh leaves of the WT and *mld1* were used in the histochemical assays. The DAB solution (1 mg·mL^−1^ 3,3′-diaminobenzidine (DAB) containing 10 mM MES (pH 6.5)) and NBT solution (0.5 mg·mL^−1^ nitro blue tetrazolium (NBT) in 10 mM potassium phosphate buffer (pH 7.8)) were used to evaluate the H_2_O_2_ accumulation and O_2_^−^ accumulation, respectively. Staining was performed as previously described [7,22].

### 4.6. Pathogen Inoculation

The inoculation strains and methods were as described previously [22]. *M. oryzae* strain H535, virulent to WT, was used to evaluate the resistance of the *mld1* plants to blast disease at the four- to five-leaf stages by the punch-inoculation method. Seedling leaves were detached and wound-inoculated with a 10 μL spore suspension (2 × 10^5^ mL^−1^) supplemented with 0.025% Tween-20. The inoculated leaves were maintained inside the incubation chamber at 28 ± 1 °C with saturated humidity in darkness for 24 h, followed by 12 h light and 12 h dark cycles. The length of the resultant lesions was measured 5 days post inoculation. For bacterial blight inoculation, *Xoo* strain PXO99 (virulent to WT) was inoculated onto the healthy flag leaves of the 8–9 week old plants using the scissor-dipping method. Scissors were dipped into the bacterial suspension (OD 0.8 in 10 mM MgCl_2_) and then used to remove the top 3 cm of the leaves. Lesion lengths were measured 2 weeks post inoculation.

### 4.7. Phylogenetic Tree

Phylogenetic analysis of *OsMLD1* and OsRpn1 was performed using sequences of their homologous sequences from monocotyledons and dicotyledons. Amino-acid sequences homologous to *OsMLD1* and OsRpn1 were downloaded from the National Center for Biotechnology Information website (http://blast.ncbi.nlm.nih.gov/, accessed on 22 April 2021). A maximum likelihood phylogenetic tree was generated with MEGA X (version 10.0.5) Analysis software using the following pipeline: bootstrap support values from 1000 pseudo-replicates of the dataset are provided as percentages at the corresponding nodes when >50% [93].

### 4.8. Subcellular Localization

The CDSs of *OsMLD1*, *OsRpn1*, *OsSERK1*, and *OsSERK2* were amplified and inserted into the pCG1301 vector to fuse in-frame with the green fluorescent protein (GFP) coding sequence, generating the 35S:OsMLD1-GFP, 35S:OsRpn1-GFP, 35S:OsSERK1-GFP, and 35S:OsSERK2-GFP fusion constructs. The expression constructs, together with mCherry-tagged organelle markers [94], were co-introduced into rice protoplasts as described previously [22]. The transformed cells were observed and photographed 16 h after infiltration through a confocal microscope (Leica TCS SP8). The transient expression construct and mCherry-tagged organelle markers were also transformed into *N. benthamiana* leaves mediated by *A. tumefaciens*. Fluorescence signals (green or red) were examined and photographed 48 h after infiltration.

### 4.9. DTT Treatment

Seeds of the WT and *mld1* were germinated in water for 3 days and then transferred to half-strength liquid Murashige Skoog (MS) medium. The developing seeds were treated by adding 2 mM DTT at 10 days after cultivation, and samples were taken after 4 hours to extract the total RNA for RT-qPCR. The primer sequences were obtained from a previous study [67,95] and are listed in Table S7. The lengths of the shoots and roots of the rice plants were calculated by ImageJ (version 1.8.0) as described previously [96] after culturing for 7 days. Equal volumes of water were added as negative controls.

### 4.10. BiFC and LCI Assays

For the BiFC assay, the CDSs of *OsMLD1* and *OsRpn1* were cloned into the p35S-SPYCE (eYFP) and p35S-SPYNE (eYFP) vectors to construct the MLD1-eYFP^C^ fusion and the OsRpn1-eYFP^N^ fusion, respectively. BiFC was performed in *N. benthamiana* leaf epidermal cells as described for subcellular localization. The eYFP signals were observed 48 h after infiltration with a laser confocal scanning microscope (Zeiss LSM 800). The primers used are listed in Table S7.

For the LCI assay, the CDS of *MLD1* was cloned into pCAMBIA-35S-Cluc to produce the MLD1-CLUC fusion, while the CDSs of OsRpn1, OsSERK1, and OsSERK2 were cloned into pCAMBIA-35S-Nluc to generate the Rpn1-NLUC, SERK1-NLUC, and SERK2-NLUC fusions, respectively. The LCI assay was performed in *N. benthamiana* leaf epidermal cells. The LUC activities were visualized 48 h post infiltration using a Tanon-5200 Chemiluminescent Imaging System (Tanon Science and Technology, Shanghai, China).

### 4.11. Co-Immunoprecipitation and SDS-PAGE Analyses

For the Co-IP assay performed in rice protoplasts, the CDSs of *OsMLD1* and *OsRpn1* were cloned into the PUC-HA and PUC-FLAG vector construct to produce the 35S:MLD1-HA fusion and 35S:Rpn1-FLAG fusion. GFP-FLAG was used as a negative control. The expression constructs were co-introduced into rice protoplasts and incubated in the incubator 28 °C for 16 h. Total protein was extracted with 1 mL of lysis buffer (50 mM Tris–HCl pH 7.5, 150 mM NaCl, 1 mM EDTA, 10% glycerol, 0.5% Nonidet P-40, 1 mM phenylmethylsulfonyl fluoride, and 1× complete protease inhibitor cocktail (Roche)). The extract was centrifuged at 12,000× *g* for 20 min at 4 °C after incubating on ice for 1 h. The supernatants (50 µL) were saved for further analysis (input fraction). Different combinations of supernatants were incubated with anti-HA agarose beads (Lablead, Beijing, China) for 3 h at 4 °C, followed by centrifugation at 2500× *g* at 4 °C for 5 min to collect immune complexes and then washed three times with wash buffer. The precipitates were released by boiling for 5 min in SDS loading buffer, and immunoprecipitated proteins were separated by 10% SDS-PAGE and analyzed by Western blot. The Co-IP assay was performed in *N. benthamiana* leaf cells as described for BiFC above. *N. benthamiana* leaves transiently co-expressing OsRpn1-GFP with MLD1-FLAG or GUS-FLAG were harvested 36 hpi. GFP-TrapA beads (Chromotek, Planegg, Germany) was used to immunoprecipitate GFP-fusion proteins from cell extracts as above. The antibodies used for Western blot included GFP (1:5000), HA (1:5000), and FLAG (1:5000). The Co-IP experiments were repeated two times independently.

### 4.12. LC–MS/MS Analyses

Rice leaves were ground to fine powder under liquid nitrogen, before adding five times the volume of lysis buffer (TCA:acetone (1:9). Total proteins were extracted and processed according to the “filter-aided sample preparation” (FASP) method as reported [73,74]. After digestion, peptides were eluted in the lectin-binding buffer. Lectin enrichment and deglycosylation were performed in H_2_^18^O for 3 h at 37 °C. The peptides were separated by chromatography and analyzed by mass spectrometry using Q-Exactive Mass spectrometer (Thermo Fisher Scientific, Waltham, MA, USA ).

The proteins and *N*-glycosites were identified with the Andromeda search engine in Max Quant software (Maxquant1.6.14). The tandem MS data obtained were searched against the UniProt *Oryza sativa* 215, 375 sequences. Max Quant search parameters were as follows: fixed modification was set as carbamidomethyl; variable modification was set as oxidation on methionine and deamidation (^18^O) in asparagine, allowing up to two missed cleavages. The maximum peptides and site false discovery rates were set at 0.01. The peptide tolerance and mass tolerance parameters for protein identification were set at 20 ppm.

The proteins annotated by Clusters of Orthologous Groups (COGs) [97] were classified into 25 COG categories. The subcellular localization of the protein was predicted with WoLF PSORT, a subcellular localization prediction program (http://wolfpsort.seq.cbrc.jp/, accessed on 14 May 2022).

### 4.13. Cell Death Detection

Cell death in the protoplasts was measured using a previously described method [98]. Briefly, protoplasts were prepared from 12 day old yellow rice seedlings. Each transformation trial contained 200 µL of protoplasts, 10 µg of test plasmid DNA, and 5 µg of pUBI:LUC plasmid DNA containing the reporter gene LUC. The empty vector was used as a negative control. The LUC activity was detected following the manufacturer’s instructions for Luciferase Assay Systems (Promega, Madison, WI, USA). The fluorescence was measured by a microplate reader (MD SpectraMax i3).

Cell death was assessed in the *N. benthamiana* leaves by *A.* *tumefaciens* with the binary plasmids 35S-SERK1 and 35S-SERK2, along with MLD1-GFP or GFP. The SERK1 and SERK2 cell suspensions were normalized to an OD_600_ of 0.1, and the MLD1-GFP and GFP cell suspensions were normalized to OD_600_ of 0.6 for co-infiltration into the leaves of *N. benthamiana*. BAX was used as a positive control. Images were taken 7 days after infiltration.

## Data Availability

The data presented in this study are available upon request from the corresponding author. The mass spectrometry proteomics data were deposited to the ProteomeXchange Consortium (http://proteomecentral.proteomexchange.org, accessed on 17 May 2022) via the iProX partner repository with the dataset identifier PXD033933.

## Supplementary Materials

The following supporting information can be downloaded at www.mdpi.com/article/10.3390/ijms23105819/s1.
