# Supplementary material for: The Rice Malectin Regulates Plant Cell Death and Disease Resistance by Participating in Glycoprotein Quality Control
Source: Int J Mol Sci. 2022 May 22;23(10):5819. doi: 10.3390/ijms23105819 (PMC9144812; doi:10.3390/ijms23105819)
Supplement: Supplementary file 1 [file ijms-23-05819-s001.zip › ijms-1724661-Supplementary.pdf]

International Journal of
Molecular Sciences

MDPI

Supplementary Materials

The Rice Malectin Regulates Plant Cell Death and Disease Resistance by Participating in Glycoprotein Quality Control

Huijing Feng [1,2], Tiancheng Qiu [1,2], Changfa Yin [1,2], Xiaosheng Zhao [1,2], Guangyuan Xu [2], Linlu Qi [2], Yan Zhang [2], Youliang Peng [1,2] and Wensheng Zhao [1,2,*]

1 State Key Laboratory of Agrobiotechnology, Beijing 100193, China
2 MOA Key Lab of Pest Monitoring and Green Management, College of Plant Protection, China Agricultural University, Beijing 100193, China; fenghj0425@163.com (H.F.); qtc303@163.com (T.Q.); yinchangfa@cau.edu.cn (C.Y.); zxs718@126.com (X.Z.); xuguangyuan@cau.edu.cn (G.X.); qilinlu1987@126.com (L.Q.); yzhang@cau.edu.cn (Y.Z.); pengyl@cau.edu.cn (Y.P.)
* Correspondence: mppzhaws@cau.edu.cn

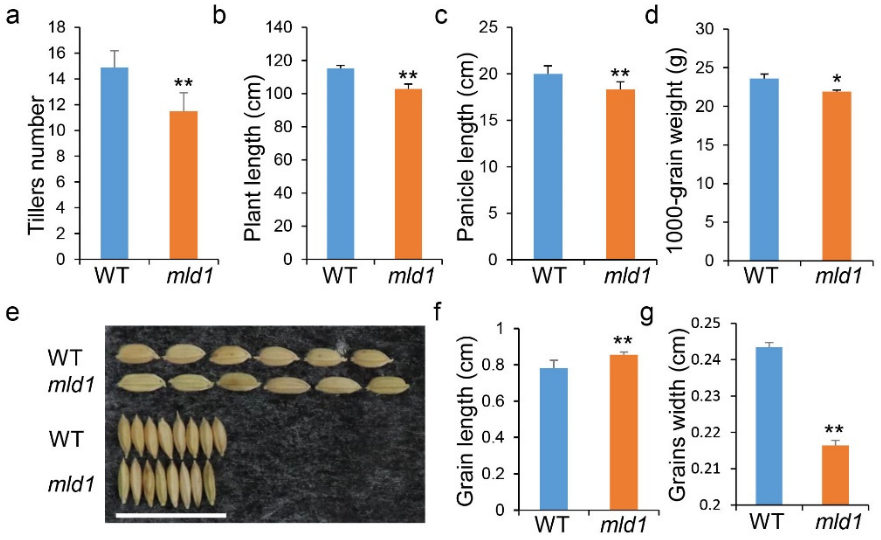

Figure S1. Several agronomic traits of WT and *mld1* plants. The *mld1* decrease tillers number (a), plant height (b), panicle length (c) and 1000-grain weight (d) compared with the WT plants. (e) Grain length and width observation of the WT and *mld1*. (Scale bar: 2 cm). The *mld1* was longer and thinner in grain length (f) and grain (g) width than WT. Data represent means ± (SD), n=6 (f), n=8 (g) (**P < 0.01, * P < 0.05; Student's *t*-test).

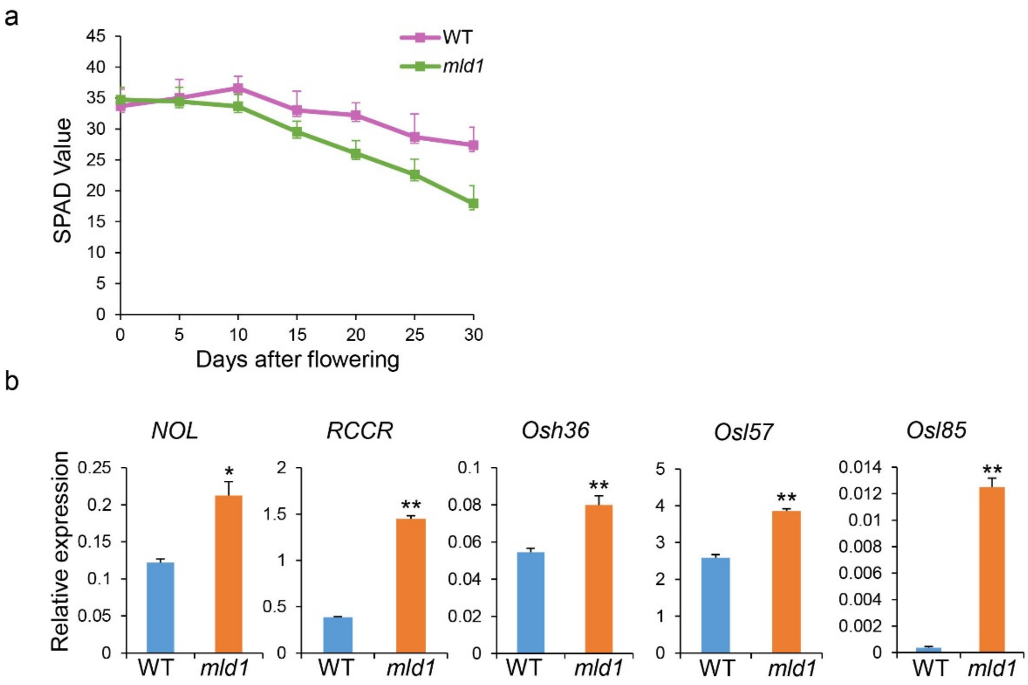

Figure S2. Senescence indicators analysis of WT and *mld1* plants. (a) Chlorophyll content for the corresponding time course indicated by the soil plant analysis development (SPAD) value. Data represent means ±SD (n=15). (b) Expression levels of senescence-related genes. Values are means ±SD (n=3) (**P < 0.01, *P < 0.05; Student's t-test).

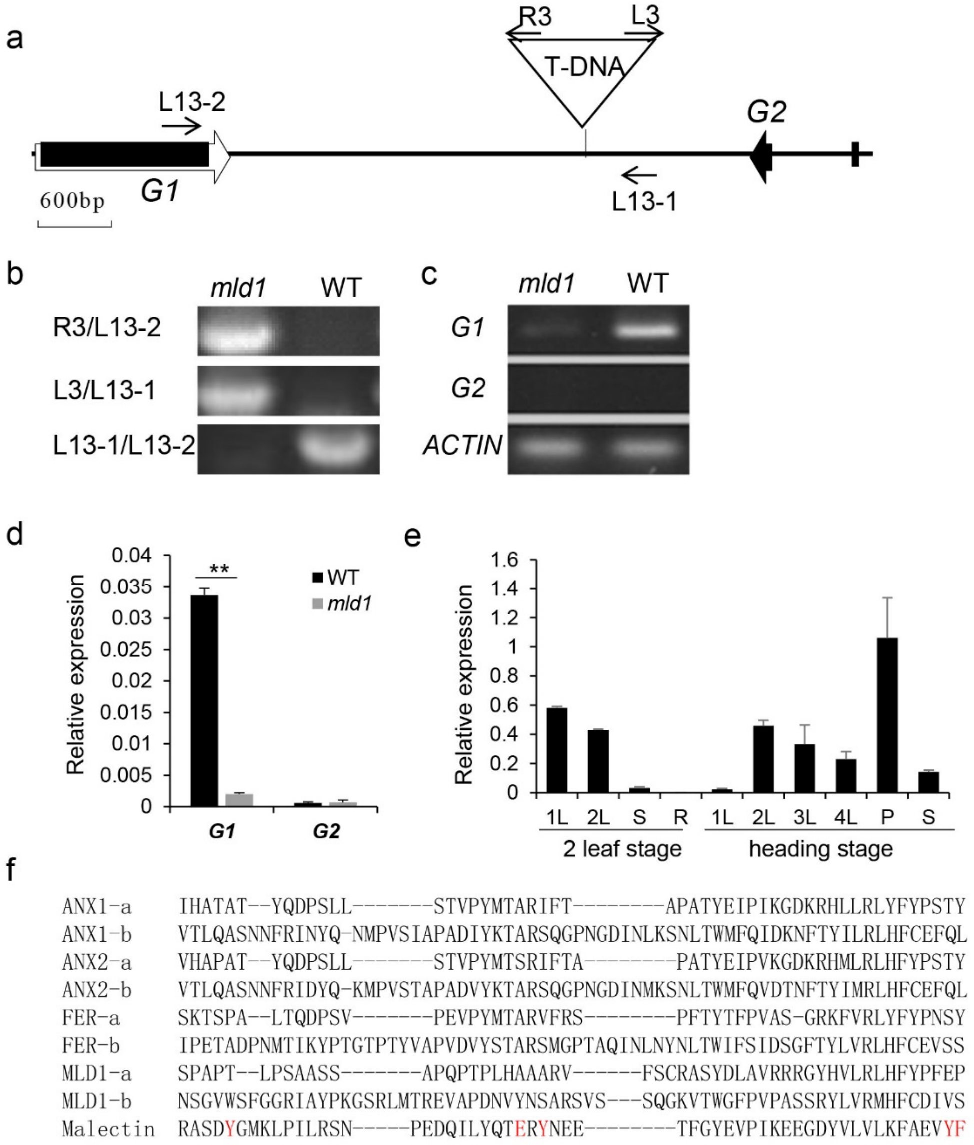

Figure S3. Molecular cloning and expression patterns of the *OsMLD1* gene. (a) Location of the T-DNA insertion in the *mld1* mutant. The triangle indicates the T-DNA insertion site in the *mld1* mutant. Black boxes indicate exons, lines indicate introns, and white boxes indicate the untranslated regions. (b) Reconfirmation of the T-DNA insertion site by special PCR. (c, d) Expression of two genes within the vicinity of the T-DNA insertion site were detected in the WT and *mld1* plants by RT-PCR (c) and RT-qPCR (d) analysis, respectively. *G1* indicates significant differences between the WT and *mld1* plants. Values are means ±SD (n=3) (**P < 0.01; Student's t-test). Total RNA was extracted from the leaves of rice 30-day seedlings. *OsACTIN1* gene was amplified as a control. (e) Expression patterns of the *LOC_03g03290* (*OsMLD1*) gene in the WT plants. Total RNA was extracted from different tissues of rice at the seeding stage and heading stage. 1L, 2L, 3L, 4L represent the first leaf, second leaf, third leaf, and fourth leaf from top to the bottom. S, R and P represent stem, roots and panicles respectively. Data were normalized to the expression of the *OsACTIN* gene. Values are means ±SD (n=3). (f) The diglucoside-binding residues in malectin are not present in any of the malectin-like domains in OsMLD1. Sequence alignment of malectin domain A and domain B from ANX1, ANX2, FER, OsMLD1 and malectin from X. laevis. Red fonts mark residues that form an NGR-perception pocket in malectin.

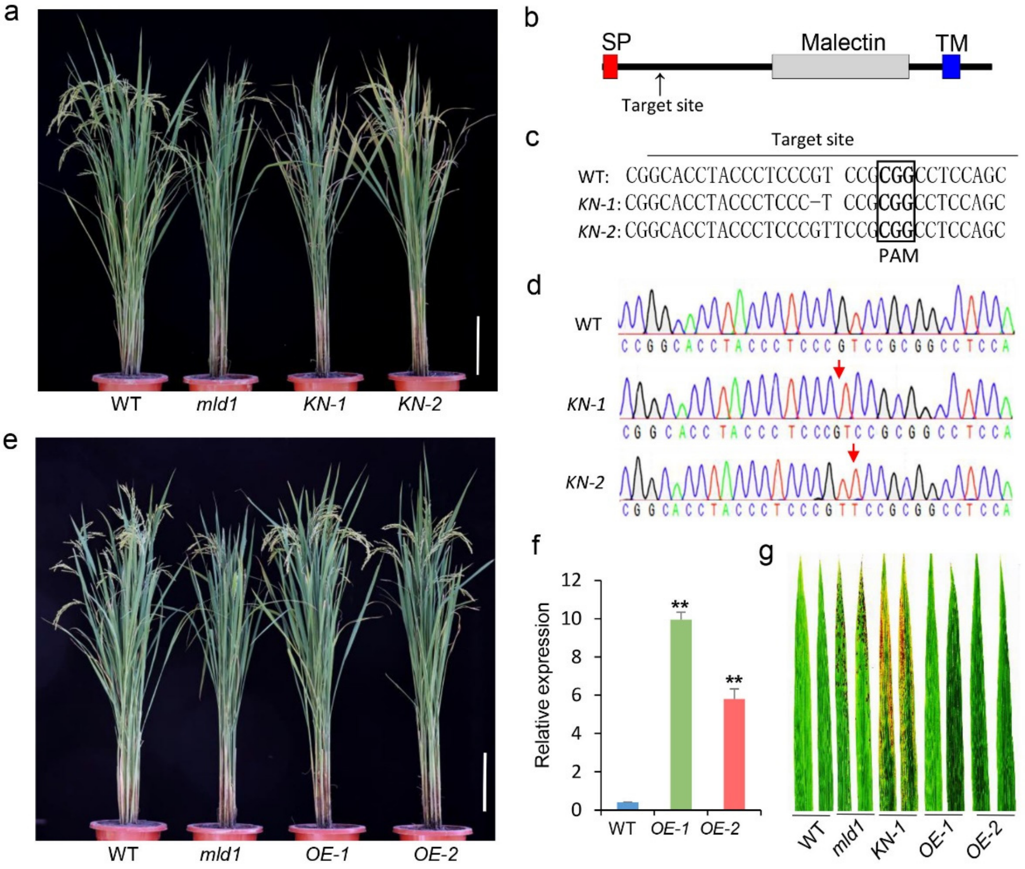

Figure S4. Phenotype of WT, *mld1*, *KN* and *OE* plants. (a, e) Whole views of two *KN* lines plants (a) and two overexpression lines plants (e) grown in filed condition at heading stage, compared with the WT and *mld1* plants. Scale bar: 20 cm. (b) Diagram of the CRISPR target site in the exon of *OsMLD1*. (c) Homozygous alleles of *KN* obtained by the CRISPR/Cas9 system. A single nucleotide deletion in *KN-1* and insertion in *KN-2* caused a frameshift and premature termination of translation. PAM, protospacer-adjacent motif sequence. (d) Sequencing analyses of the CRISPR target site in WT, *KN-1*, and *KN-2* transgenic lines. Red arrows indicate the base deletions. (f) Expression of *OsMLD1* was detected in the WT, *mld1*, *OE-1*, *OE-2* plants by RT-qPCR. Values are means ±SD (n=3) (**P < 0.01; Student's *t*-test). *OsACTIN1* gene was amplified as a control. (g) Leaves phenotypes of the WT, *mld1*, *KN* and *OE* plants at the 90 day in field conditions.

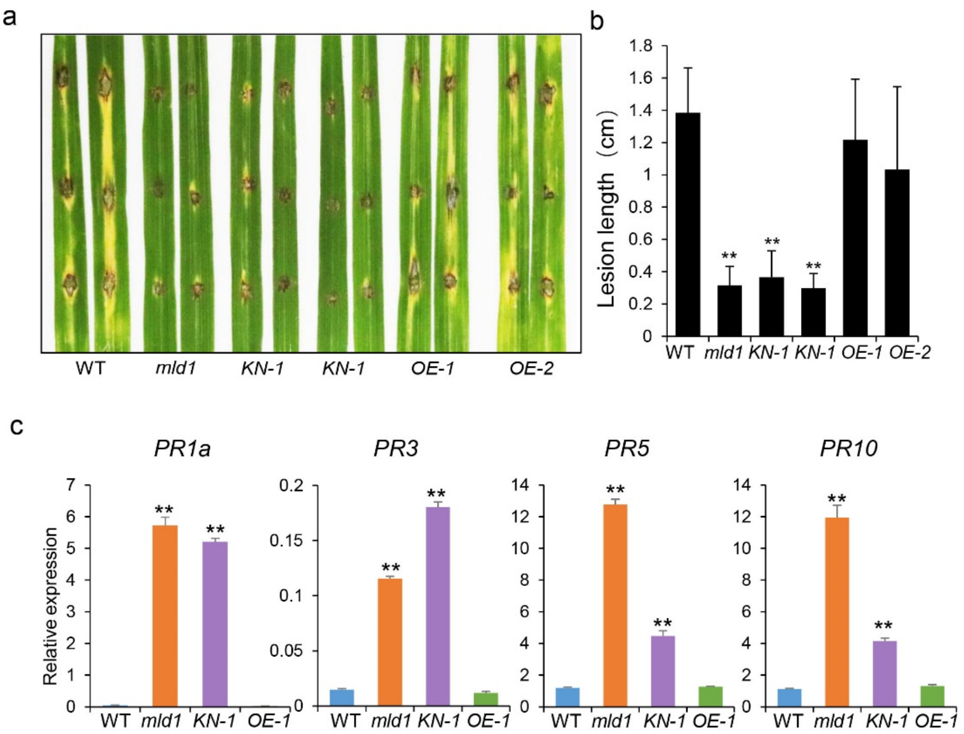

Figure S5. The *KN* plants showed enhanced resistance to *M. oryzae* and elevated expression of pathogenesis-related genes. (a, b) Punch inoculation of the WT, *mld1*, *KN-1*, *KN-2*, *OE-1*, *OE-2* plants with the compatible *M. oryzae* isolate H535. Leaves phenotype (a) and lesion length (b) at 120 h after inoculation. Data are the means ±SD (*n*=6). (c) Expression of defense marker genes in the WT, *KN* and *OE* plants (*OsACTIN1* as a reference gene). Values are means ±SD (*n*=3) (**P < 0.01; Student's *t*-test).

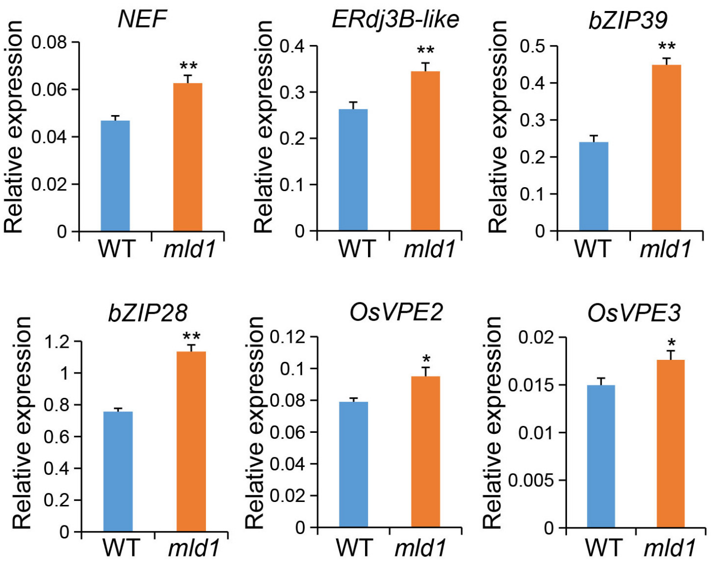

Figure S6. Expression analysis of ER-stress induced genes in the WT and *mld1* mutant plants based on RT-qPCR. *OsACTIN1* as a reference gene. Values are means ±SD (*n*=3) (*P < 0.05, **P < 0.01; Student's *t*-test).

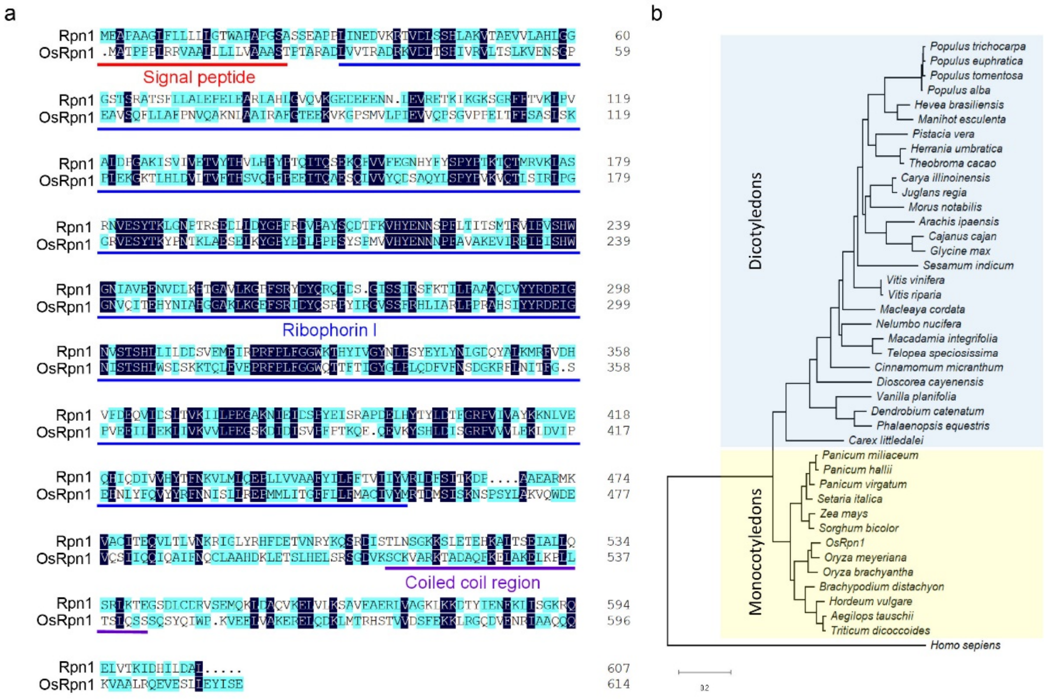

Figure S7. Sequences alignment and phylogenetic analysis of OsRpn1 and its homolog from *Homo sapiens* (AAH10839.1) and other plants. (a) Sequence alignment and phylogenetic analysis of OsRpn1 and its homolog from *Homo sapiens* (AAH10839.1) (b) Phylogenetic tree of OsRpn1 and its homologs from other plant species. *Oryza sativa* Geng, OsRpn1; *Oryza meyeriana* var. *granulata*, KAF0935191.1; *Oryza brachyantha*, XP_006654235.2; *Panicum miliaceum*, RLN18009.1; *Brachypodium distachyon*, XP_003558910.1; *Setaria italica*, XP_004985866.1; *Panicum hallii*, XP_025792675.1; *Panicum virgatum*, XP_039780175.1; *Zea mays*, PWZ54446.1; *Hordeum vulgare*, KAE8799716.1; *Aegilops tauschii* subsp. *strangulata*, XP_020198000.1; *Sorghum bicolor*, XP_002465935.1; *Triticum dicoccoides*, XP_037426547.1; *Macadamia integrifolia*, XP_042495731.1; *Telopea speciosissima*, XP_043712880.1; *Cinnamomum micranthum*, RWR78537.1; *Carex littledalei*, KAF3321095.1; *Dendrobium catenatum*, XP_020704065.1; *Phalaenopsis equestris*, XP_020599381.1; *Pistacia vera*, XP_031270275.1; *Vitis vinifera*, XP_002274236.1; *Vanilla planifolia*, KAG0475986.1; *Carya illinoinensis*, XP_042989052.1; *Populus alba*, XP_034931404.1; *Morus notabilis*, XP_024020135.1; *Vitis_riparia*, XP_034676405.1; *Herrania umbratica*, XP_021285467.1; *Populus trichocarpa*, XP_002307811.1; *Sesamum indicum*, XP_011100198.1; *Populus euphratica*, XP_011041178.1; *Theobroma_cacao*, EOY15338.1; *Juglans regia*, XP_035551581.1; *Macleaya cordata*, OVA00654.1;*Cajanus cajan*, XP_020219287.1; *Dioscorea cayenensis* subsp. *rotundata*, XP_039135074.1; *Hevea brasiliensis*, XP_021666095.1; *Manihot esculenta*, XP_021600999.1; *Populus tomentosa*, KAG6774850.1; *Glycine max*, XP_003527283.1; *Nelumbo nucifera*, XP_010266249.1; *Arachis ipaensis*, XP_016167084.1 and *Homo sapiens*, AAH10839.1. The phylogenetic tree was created using the maximum likelihood method in MEGA X software. Bootstrap support values from 1000 pseudoreplicates of the dataset are provided as percentages at the corresponding nodes when >50%.

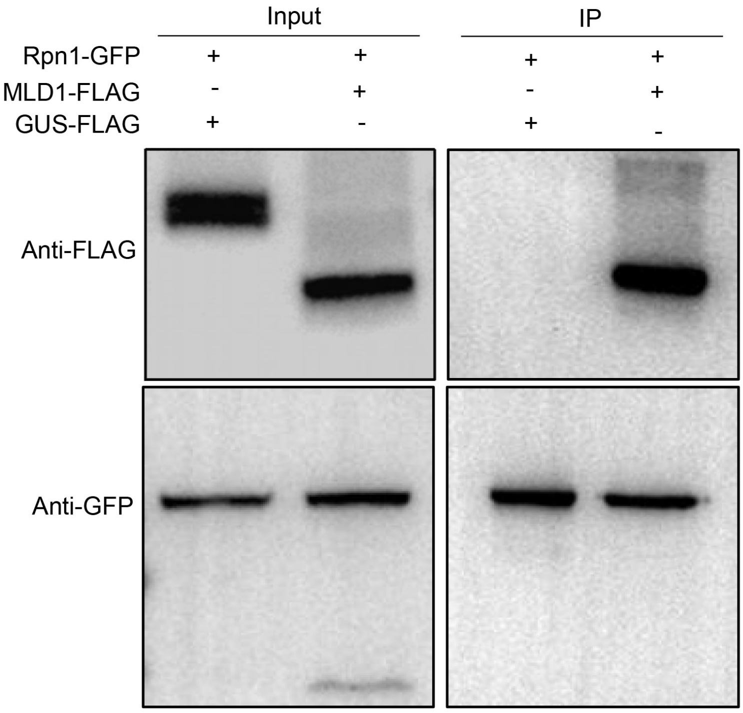

Figure S8. Co-IP assays for the interactions of OsMLD1 with OsRpn1 in *Nicotiana benthamiana*. Rpn1-GFP (~110 kD), MLD1-FLAG (~50 kD) and GUS-FLAG (~ 75 kD) were expressed in *N. benthamiana.* Co-IP was performed using GFP-binding beads. Proteins were detected with antibodies as indicated. Similar results were obtained in three independent experiments.

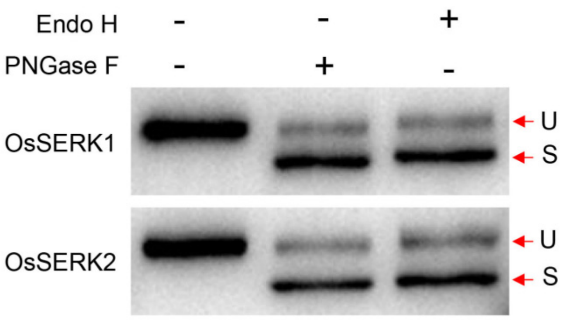

Figure S9. Western blot analysis showed OsSERK1 and OsSERK2 were glycoproteins in *Nicotiana benthamiana.* The glycans were cleaved from OsSERK1 or OsSERK2 by PGase F or Endo H. Red arrow indicates uncleavaged (U) or cleavaged.

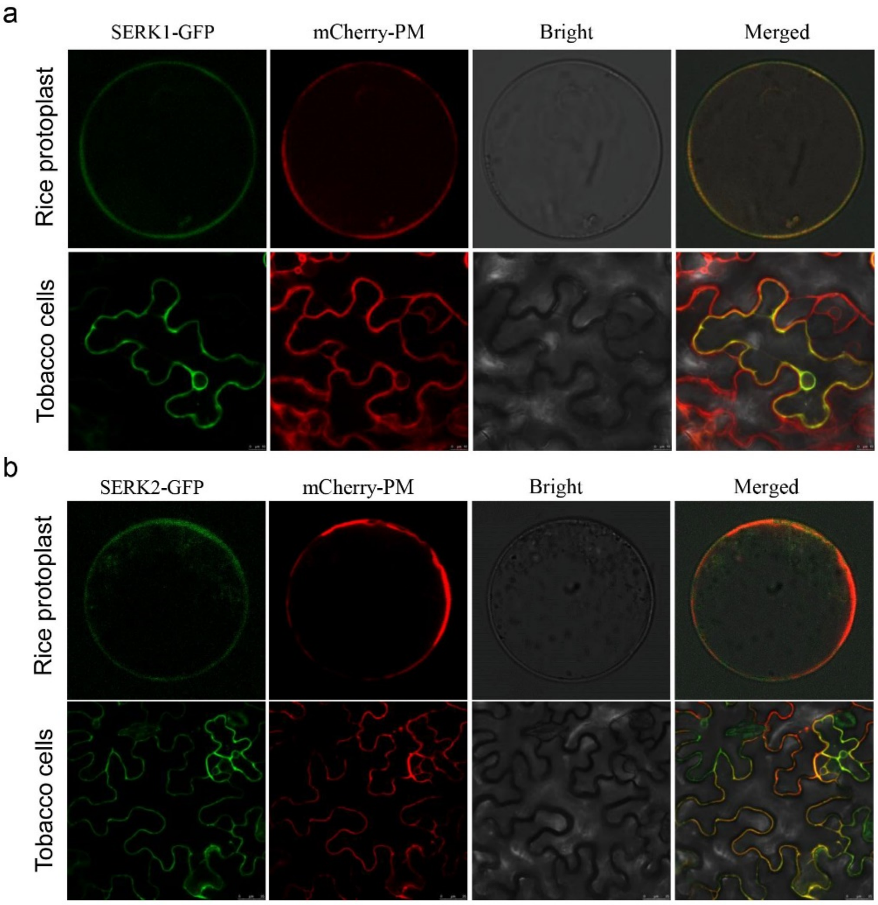

Figure S10. OsSERK1 and OsSERK2 are membrane localization proteins. Subcellular localization of OsSERK1 (a) and OsSERK2 (b) in rice protoplasts and *Nicotiana benthamiana* leaves. Co-expression of OsSERK1-GFP or OsSERK2-GFP and mCherry-PIP2A (PM) in the rice protoplasts and the epidermal cells of *N. benthamiana*.

Supplementary Tables

Table S1 Genetic analysis of *mld1*.xls.

Table S2 Identified N-glycosites and N-glycoprotein groups.xls.

Table S3 Identified peptides length in *mld1* and WT.xls.

Table S4 Motif analysis of identified peptides.xls.

Table S5 COG enrichment of identified N-glycoproteins in *mld1* and WT.xls.

Table S6 Subcellular location of *N*-glycoproteins in *mld1* and WT.xls.

Table S7 Primers used in this study.xls.
