# Supplementary material for: The Rice Malectin Regulates Plant Cell Death and Disease Resistance by Participating in Glycoprotein Quality Control
Source: Int J Mol Sci. 2022 May 22;23(10):5819. doi: 10.3390/ijms23105819 (PMC9144812; doi:10.3390/ijms23105819)
Supplement: Supplementary file 1 [file ijms-23-05819-s001.zip › Table S1 Genetic analysis of mld1.pdf]

Table S1 Genetic analysis of *mld1*

class	T1 plants	T2 HygB resistance homozygous plants	T2 HygB resistance separation plants	T2 HygB susceptible plants
Mutant phenotype	4	4	0	0
WT phenotype	18	0	12	6
total	22	4	12	6
