# Supplementary material for: The Rice Malectin Regulates Plant Cell Death and Disease Resistance by Participating in Glycoprotein Quality Control
Source: Int J Mol Sci. 2022 May 22;23(10):5819. doi: 10.3390/ijms23105819 (PMC9144812; doi:10.3390/ijms23105819)
Supplement: Supplementary file 1 [file ijms-23-05819-s001.zip › Table S3 Identified peptides length.pdf]

Table S3 Identified peptides length

**peptides in WT**	**length (acids)**	**peptides in *mld1***	**length (acids)**
DLWINKL	7	INVSNPR	7
AGLTKNR	7	NTNITAK	7
PQGSNKR	7	NECMSFR	7
ESGLNLNPK	9	PQGSNKR	7
MMMINTQCNR	10	ESGLNLNPK	9
TANNTEPFNR	10	LNSLNLSSNR	10
LDNGNHMVSANK	12	QDANVSEIKK	10
PQHKNAEPMVSR	12	MNVSQSMPEK	10
AEYEQMMNNLHN	12	QLINLSLSENK	11
ATANDISNPNGK	12	IFLFNNATNVR	11
ALSFLPNFGNYKR	13	ASMEPLNRMVR	11
MLTVAASALRNNK	13	SNETGIAGFGR	11
ANAENKALSGDNK	13	TSSAESGNVSR	11
FLSDLNLAMADMK	13	IGISAFMNSKR	11
LDSEIFFAPNSNSI	14	DGRDNLCLVDK	11
DENMAGEWNHTSGK	14	IIRSTNSDQNR	11
DAIEGMNGKELDGR	14	LNNNSLSGSIPK	12
YLNMGDVNLSMADYR	15	LGMVDMSSNGAR	12
ESAQDIINLKPLIEK	15	SLTSLTSLNLSR	12
KCGPHEDNFGLVEPR	15	FESLVNGGNCSR	12
SAAAAASNVEFIRSR	15	YGIQTNSTPVAR	12
DFKTFVNVETNPNTNR	16	GNNIAHSNTSCR	12
VLDSVNITLGAEDELK	16	YINKPSGNANTK	12
MLDATFNAKLGDFGLAR	17	HEDHYLNKYANK	12
MASMKLVADNGATSTTK	17	TIAASIMPESNR	12
GGVSGNGGMAMAAVATS	17	LQNLTQYQAVER	12
MLAGVVMPNKSSSNSSR	17	AEYEQMMNNLHN	12
METVSEASMGHGMNYALK	18	ATANDISNPNGK	12
NRNNIHDPNFFRCQCSSK	18	GLNISDDDYDSPR	13
GGLDFTKDDENVNSQPFMR	19	SDQGLIEDAQTNR	13
RQISMFDNLALAFQNSGTK	19	VSGEVANLCNALR	13
EEKVNDSSKNAENADNPIEK	20	MDNCTNIANVYPK	13
QLLDLDSLSFHQGGLLMANKK	21	VMIQSTNSSVLVK	13
IAVGELQHCNLSYNIAVVNVK	21	MLSGLDDGLENRR	13
GNIPTIFLHSVLPNLLENPEFQNRR	25	SAMDWGINGGLGR	13
ATCAADLAPLLGPVAANATDYLCNR	25	MLTVAASALRNNK	13
FSTLVPGATSFPQPILTAASFNASLFR	27	ANAENKALSGDNK	13
LIIQSNIYNYLGILLEGRERFEEEAIADR	29	FLSDLNLAMADMK	13
PLDGNAETQVSSTSPMHVPVISIVGPGG MGK	31	AVLPDSNVTVASQK	14

SPATSLKQLPCVSLSSSMMEENQSVTHGNQQSINVDWNK	39	NPDPNPPRPNYSRR	14
FLGNISMLSYLNLSFNNFSGEVPDFGVFTNITAFLIQGNDK	41	INNTNLEFFPVKNK	14
		LTDAVTNNGMSSRK	14
		MKAATGVAADEVNR	14
		LDSEIFFAPNSNSI	14
		DENMAGEWNHTSGK	14
		DAIEGMNGKELDGR	14
		TRPDIDGLNGTQSNR	15
		GENTGDNPNMSILQR	15
		SAAAAASNVEFIRSR	15
		LAPGVQNITTVDEAEK	16
		TIDLEQGTIYNMCTQK	16
		ILLGWANESDSVTYDK	16
		GVCQTGDAFNTTNCNR	16
		LHLASISAQPNPWCKK	16
		QLAALLENNAESSAIR	16
		VLDSVNITLGAEDELK	16
		VERYNLDVDECISSNPR	17
		MDMALPIVNATAAVLAR	17
		NDLGIENTYPWTIMTDK	17
		GGVSGNGGMAMAAVATS	17
		MLAGVVMPNKSSSNSSR	17
		TSNLTALSEQQLVDCDTK	18
		ALHQGNRTVTEYLHEFDR	18
		EDLENVAGMGSATASGGK	18
		MKSLQTLINNVQVLQEASK	19
		HCNCKNSQCLKLQEAITVR	19
		VTSPAGATRCVDAANDLAR	19
		IPAATGSGNKENNISQNRR	19
		LPQEAKNKDQGLCSSNGPR	19
		RQISMFDNLALAFQNSGTK	19
		MRRNTVLNLGVEEGGNHGYR	20
		INQSPNVLNQAMHSERTQDK	20
		YINLTFGCPLLDTVVDDLTIK	21
		MIGHNNDEFDDQSISNTETTK	21
		TNSILSAQSTTGSFNSTNSDR	21
		HLFGNTAAAAPAADSYNPKNK	21
		IAVGELQHCNLSYNIAVVNVK	21
		METDDAPNEAASGTDVNMQEAK	22
		LPDSVCEDLTRISRNFWWGAENR	23
		GVAEALNETTCFDNQCEGLVIQGK	24
		ATCAADLAPLLGPVAANATDYLCNR	25

	SIGHINNPVYSSDIHNEVGRFTSVEK	26
	SLGVLNLSHNSLSGTIPTTLNDLPVMSK	28
	IGHESDLFVGNALMDFYAKCNDMDSSLK	28
	RDYSLFVNIYFTFTNMSNTGDNGNTGYK	28
	NRNPPIGSNGDNGGNGDGSGGDGGGRSSK	29
	AAAAAPVENSADKGPHQDSQPPSAAAPAK	29
	AQDQQQQVKEEEEEAAVENLPPPPQEEEQR	30
	DSTTASEALANTDLPAPSSSLAELIGNFSR	30
	RDTVMYKVSVQMIEIYNEQIHDLLGNSGLE<br>NR	32
	FVVGSTNASWQTNDHSGAASHDAAAAAPA<br>AAVLGGGHGVPR	41
