# Supplementary material for: The Rice Malectin Regulates Plant Cell Death and Disease Resistance by Participating in Glycoprotein Quality Control
Source: Int J Mol Sci. 2022 May 22;23(10):5819. doi: 10.3390/ijms23105819 (PMC9144812; doi:10.3390/ijms23105819)
Supplement: Supplementary file 1 [file ijms-23-05819-s001.zip › Table S4 Motif analysis of identified peptides.pdf]

Table S4 Motif analysis of identified peptides

Motif	Motif Logo	Score	Number of peptides	Foreground Matches	Background Matches	Fold Enrichment	*p*-value
N-X-T	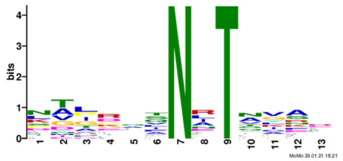	3.56	23	23/169	22359/365844	2.2	0.00028
N-X-S	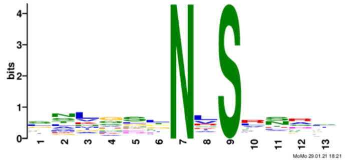	4.13	30	30/169	33618/365844	1.9	0.00036
N-X-X-N	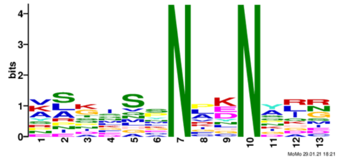	2.82	18	18/169	15909/365844	2.4	0.00045

occurrences = 15, significance = 0.01, background = Ensembl *Oryza sativa*: 43
