# Supplementary material for: The Rice Malectin Regulates Plant Cell Death and Disease Resistance by Participating in Glycoprotein Quality Control
Source: Int J Mol Sci. 2022 May 22;23(10):5819. doi: 10.3390/ijms23105819 (PMC9144812; doi:10.3390/ijms23105819)
Supplement: Supplementary file 1 [file ijms-23-05819-s001.zip › Table S6 subcellular location of N-glycoproteins.pdf]

Table S6 subcellular location of *N*-glycoproteins

Subcelluar	number			percentage		
	mld1	WT	shared	*mld1*	WT	shared
chloroplast	28	4	7	38.89	16.67	41.18
nuclear	15	6	7	20.83	25	41.18
plasma membrane	10	4	1	13.89	16.67	5.88
cytoplasmic	9	8	1	12.5	33.33	5.88
extracellular	6	1	0	8.33	4.17	0
endoplasmic reticulum	2	0	0	2.78	0	0
Golgi body	1	0	0	1.39	0	0
vacuolar	1	1	1	1.39	4.17	5.88
All	72	24	17	100	100	100
