# Supplementary material for: The Rice Malectin Regulates Plant Cell Death and Disease Resistance by Participating in Glycoprotein Quality Control
Source: Int J Mol Sci. 2022 May 22;23(10):5819. doi: 10.3390/ijms23105819 (PMC9144812; doi:10.3390/ijms23105819)
Supplement: Supplementary file 1 [file ijms-23-05819-s001.zip › Table S7 primers used in this study.pdf]

Table S1 primers used in this study

Primer name	Forward primer (5'-3')	**Reverse primer (5'-3')**	**Enzyme site**	**Use of primers**
L13-1	GATATGCCCACCCTTTACTCA			Identification for T-DNA insertion site
L13-2	ATGTCAATGGTCAGCTAGCAGT			Identification for T-DNA insertion site
L3	GATGCCGACCGGATCTGTCGATC			Identification for T-DNA insertion site
R3	CTGTTGCCGGTCTTGCGATGAT			Identification for T-DNA insertion site
G1(LOC_Os03g03290)	TCTTCTCCTGCCGCGCGTCCTA	TGAGACGACA ATCCATTGGT		RT-PCR
G2(LOC_Os03g03300)	ATGGAGGGAGCTAGCAATGGA	TTAGTATGTC CGAATGTC		RT-PCR
ACTIN	TGGAACTGGTATGGTCAAGGC	AGTCTCATG GATACCCGCAG		RT-PCR
G1(LOC_Os03g03290)	GTGGATGTTGAGGATGAAGGGGCC	TCTTAGCGGACACAAGTTGATATGCTC		qRT-PCR
G2(LOC_Os03g03300)	GCTAGCAATGGAGCATTTCTTCCCTG	TTAGTATGTCCGAATGTCGTTACTGCCG		qRT-PCR

OsRAc1(LOC_Os03g50885)	ATCACTGCCTTGGCTCCTA	CATCTGCTGGAATGTGCTG		qRT-PCR
OsPR1a(LOC_Os01g28450)	CGTCGGACAGAGGCCTTACT	GACCGTGAAGGCGTGGAT		qRT-PCR
OsPR1b(LOC_Os07g03710)	GGCAACTTCGTCGGACAGA	CCGTGGACCTGTTTACATTTTCA		qRT-PCR
OsPR3(LOC_Os10g39680)	CCTATTGCATGATCGTTCGAT	GCCTGTAGCAGTTAAAGCAATTG		qRT-PCR
OsPR5(LOC_Os12g43380)	CCACGTGTGCAATTGTTTAATC	ACTCGGACGCTTTCATTTGA		qRT-PCR
OsPR8(LOC_Os10g28080)	TTCATCTGGTCAGCGGATAGC	TATCACGACCGTTCGATGGA		qRT-PCR
OsPR10 (LOC_Os12g36880)	CCTGCCGAATACGCCTAAGA	CTCAAACGCCACGAGAATTT		qRT-PCR
WRKY45(LOC_Os05g25770)	AGCAATCGTCCGGGAATTC	GAAGTAGGCCTTTGGGTGCTT		qRT-PCR
WRKY62(LOC_Os09g25070)	TGAAGGATGGGTACCAATGGA	CACATCTTTGGAGCTTCTTCTTGA		qRT-PCR
NOL(LOC_Os03g45194)	CCACGAAAGGTATAGGATATG	TCAAGTCAGTCACCGCAGAT		qRT-PCR
RCCR1(LOC_Os10g25030)	CGCATTTCCTCATGGAATTT	CTTCTCACGCTGTTTGTCCA		qRT-PCR
Osh36(LOC_Os05g39770)	GCACGGAGGCGAACGA	TTGAGCGGTAGCACCCATT		qRT-PCR
Osl57(LOC_Os02g57260)	ACCCTAAAGTAAATGAAGTC	CCTGCTCTTGTCTTGTTA		qRT-PCR
Osl85(LOC_Os07g34520)	GAGCAACGGCGTGGAGA	GCGGCGGTAGAGGAGATG		qRT-PCR
3Flag+OsMLD1	TACTGCAGCACCAATCCGCCAT	ATACTAGTAGGTCAACAGCCACAGT	Pst I / Spe I	pMLD1-OE vector construction

CRISPR-OsMLD1	ggcaGGCACCTACCCTCCCGTCCG	aaacCGGACGGGAGGGTAGGTGCC	Bsa I	MLD1-KN vector construction
OsMLD1-GFP	ATAGGATCCATGGCTAGTGTTCTCG	ATAGTCGACATAGTCTGTCTTAGCGGAC	Bam HI / Sal I	p35S:GFP-MLD1 for subcellular localization
Hyr	GCTGCGCCGATGGTTTCTACAA	CACGGCCTCCAGAAGAAGATGTTG		Transgenic plant test
MLD1-KN -JD	CCACACCAATCCGCCATG	CGAAGGTGAGGACGAGGAC		Identification for KN site
OsbZIP60(LOC_Os07g44950)	TGGTTATTAAGTCCATTGTTGCGAGTG	AGTCTCCTCCTTTCCTTGGTTTCC		qRT-PCR
OsbZIP39(LOC_Os05g34050)	CCAAGGGAGGCTGGTAATG	AAAGGAAGCGTGCAGGAGTA		qRT-PCR
OsbZIP28(LOC_Os03g20310)	TATCACAGGGAATGGAAGC	GATGTAAAGTCTGCTGCCG		qRT-PCR
OsbZIP50(LOC_Os06g41770)	CGCCAGAGCTTGTTGAAGGATAGG	GCGGCAGGGTTTCCGTGAGTA		qRT-PCR
OsCNX(LOC_Os04g32950)	GAAGGACGACTACAAAGGTG	ACTCAAGGCCATTCTGAAG		qRT-PCR
OsCRT1(LOC_Os07g14270)	AATGGAAGGCACCGATGA	GTCTCCTCTGCGAATGTCT		qRT-PCR
OsCRT2(LOC_Os03g61670)	GAGGATGAGGCGGACGATGA	CCTTGATGTCCTGCGGTTTC		qRT-PCR
OsIRE1(LOC_Os07g28820)	TGCCCTGCTTGCAGACATGG	GACCCAAGTAGCTCCTTGAGATC		qRT-PCR
NEF(LOC_Os09g33780)	ATTAAAGATGCCGTCAGATGCT	GTCAATGGGCTCAACGAGAAT		qRT-PCR
OsERdj3B(LOC_Os05g06440）	AACTGTTACAATCTCCCTGCTC	GGTGCCAATTTCCACCATA		qRT-PCR

OsVPE2(LOC_Os08g06010)	CGGCTCCAACGGCTACTACAAC	TCGGGACCCCAGCATAGACA		qRT-PCR
OsVPE3(LOC_Os02g43010)	CGGTAACTACAGGCACCAGGC	GTGACTTCGTCTCCAGTGTAATCC		qRT-PCR
OsbZIP50 splice	CCAGAGCTTGTTGAAGGATAG	GGTTTCGGTTGGGTAGAC		RT-PCR
NYFP-Rpn1	ATTAACAAGGCCATTACGGCCATGGCG ACGCCACCGCCG	AACTGATTGGCCGAGGCGGCCCCGG ATCTCACTGATGTACTCT	Sif I	BiFC assay
CYFP-MLD1	ATTAACAAGGCCATTACGGCCATGGCT AGTGTTCTCGCCGTC	AACTGATTGGCCGAGGCGGCCCCGT CAATAGTCTGTCTTAGC	Sif I	BiFC assay
MLD1F-CLUC	ACGGGGGACGAGCTCGGTACCATGGCT AGTGTTCTC	CGCGTACGAGATCTGGTCGACATAG TCTGTCTTAGCGG	Kpn I / Sal I	LCI assay
Rpn1F-NLUC	ACGGGGGACGAGCTCGGTACCATGGC GACGCCACCGCCGCT	AACATCGTATGGGTAGTCGACGATC TCACTGATGTACTCT	Kpn I / Sal I	LCI assay
MLD1N-CLUC	ACGGGGGACGAGCTCGGTACCATGGCT AGTGTTCTC	CGCGTACGAGATCTGGTCGACTTTGT TCATCCTCATTATCTCTACAGC	Kpn I / Sal I	LCI assay
MLD1C-CLUC	ACGGGGGACGAGCTCGGTACCACAAA TGGTGGTATTGATGGGG	CGCGTACGAGATCTGGTCGACATAG TCTGTCTTAGCGGACAC	Kpn I / Sal I	LCI assay
Rpn1N-NLUC	ACGGGGGACGAGCTCGGTACCATGGC GACGCCACCGCCGCT	AACATCGTATGGGTAGTCGACATTAT GCTCT GGAATAACAT	Kpn I / Sal I	LCI assay
Rpn1C-NLUC	ACGGGGGACGAGCTCGGTACCCTATAT TTCC AGGTTTACTA	AACATCGTATGGGTAGTCGACGATC TCACTGATGTACTCT	Kpn I / Sal I	LCI assay

Rpn1-GFP	ATAGGATCCATGGCGACGCCACCGC	ATAGTCGACGATCTCACTGATGTAC	Bam HI / Sal I	Co-IP assay in *N.benthamiana*
MLD1-FLAG	TCTGATCAAGAGACAGGATCCATGGCT AGTGTTCTC	GTAGTCAGCGGCCGCTCTAGAATAG TCTGTCTTAGCGGACAC	Bam HI / Xba I	Co-IP assay in *N.benthamiana*
MLD1-HA	CGGGGGACGAGCTCGGTACCATGGCTA GTGTTCTC	ACATCGTATGGGTAGTCGACATAGT CTGTCTTAGCGGAC	Kpn I /Bgst II	Co-IP assay in rice protoplasts
Rpn1-FLAG	CGGGGGACGAGCTCGGTACCATGGCG ACGCCACCGCCG	TGGTCTTTGTAGTCTTCGAAGATCTC ACTGATGTACTCT	Kpn I /Bgst II	Co-IP assay in rice protoplasts
SERK1-NLUC	ACGGGGGACGAGCTCGGTACCATGGC GGCGCATCGGTGGGCGGT	AACATCGTATGGGTAGTCGACCCTC GGCCCTGATAGCTC	Kpn I / Sal I	LCI assay
SERK2-NLUC	ACGGGGGACGAGCTCGGTACCATGGC GGAGG CGCGGCTGC	AACATCGTATGGGTAGTCGACCCTC GGGCCGGACAGCTC	Kpn I / Sal I	LCI assay
35S-SERK1	ATACACCAAATCGACTCTAGAATGGCG GCGCATCGG	GGGAAATTCGAGCTCGGTACCTCAC CTCGGCCCTGATAG	Xba I / KpnI	cell death
35S-SERK2	ATACACCAAATCGACTCTAGAATGGCG GAGG CGCGGCTGC	GGGAAATTCGAGCTCGGTACCCCTC GGGCCGGACAGCTC	Xba I / Kpn I	cell death
SERK1-FLAG	TCTGATCAAGAGACAGGATCCATGGCG GCGCATCGGTGGGCGGT	GTAGTCAGCGGCCGCTCTAGACCTC GGCCCTGATAGCTC	Bam HI / Xba I	*N*-glycosylation modification assay

SERK2-FLAG	TCTGATCAAGAGACAGGATCCATGGCG GAGG CGCGGCTGC	GTAGTCAGCGGCCGCTCTAGACCTC GGGCCGGACAGCTC	Bam HI / Xba I	*N*-glycosylation modification assay
SERK1-GFP	GGGGTACCATGGCGGCGCATCGGTGGG CGGT	GCTCTAGACCTCGGCCCTGATAG CTC	KpnI / Xba I	
SERK2-GFP	GGGGTACCATGGCGGAGG CGCGGCTGC	GCTCTAGACCTCGGGCCGGACAG CTC	KpnI / Xba I	
